# The characteristics of mRNA m^6^A methylomes in allopolyploid *Brassica napus* and its diploid progenitors

**DOI:** 10.1093/hr/uhac230

**Published:** 2022-10-11

**Authors:** Zeyu Li, Mengdi Li, Xiaoming Wu, Jianbo Wang

**Affiliations:** State Key Laboratory of Hybrid Rice, College of Life Sciences, Wuhan University, Wuhan 430072, Hubei, China; State Key Laboratory of Hybrid Rice, College of Life Sciences, Wuhan University, Wuhan 430072, Hubei, China; Key Laboratory of Resource Biology and Biotechnology in Western China, Ministry of Education, College of Life Sciences, Northwest University, Xi’an 710069, China; Key Laboratory of Biology and Genetic Improvement of Oil Crops, Ministry of Agriculture, Oil Crops Research Institute of CAAS, Wuhan 430062, China; State Key Laboratory of Hybrid Rice, College of Life Sciences, Wuhan University, Wuhan 430072, Hubei, China

## Abstract

Genome duplication events, comprising whole-genome duplication and single-gene duplication, produce a complex genomic context leading to multiple levels of genetic changes. However, the characteristics of m^6^A modification, the most widespread internal eukaryotic mRNA modification, in polyploid species are still poorly understood. This study revealed the characteristics of m^6^A methylomes within the early formation and following the evolution of allopolyploid *Brassica napus*. We found a complex relationship between m^6^A modification abundance and gene expression level depending on the degree of enrichment or presence/absence of m^6^A modification. Overall, the m^6^A genes had lower gene expression levels than the non-m^6^A genes. Allopolyploidization may change the expression divergence of duplicated gene pairs with identical m^6^A patterns and diverged m^6^A patterns. Compared with duplicated genes, singletons with a higher evolutionary rate exhibited higher m^6^A modification. Five kinds of duplicated genes exhibited distinct distributions of m^6^A modifications in transcripts and gene expression level. In particular, tandem duplication-derived genes showed unique m^6^A modification enrichment around the transcript start site. Active histone modifications (H3K27ac and H3K4me3) but not DNA methylation were enriched around genes of m^6^A peaks. These findings provide a new understanding of the features of m ^6^A modification and gene expression regulation in allopolyploid plants with sophisticated genomic architecture.

## Introduction

More than 70% of angiosperms have experienced at least one polyploidization event in their evolutionary lineages [1]. Compared with diploid progenitors, polyploids have physiological and phenotypic adaptive advantages [2]. Polyploidization causes multilevel genetic changes, including genetic composition and gene expression, increasing adaptability through the formation of new regulatory pathways [3]. Depending on whether the genetic progenitors are the same species, polyploidy gives rise to autopolyploidy or allopolyploidy [1]. As the most common form of polyploidy, allopolyploidy has played an essential role in the evolutionary adaptation of plant species [4]. Although whole-genome duplication (WGD) provides numerous raw materials for the evolution of plants, it is episodic, and successive WGD events are spaced dozens of millions of years apart [5]. Therefore, plants need several kinds of single-gene duplication, including tandem duplication (TD), transposed duplication (TRD), dispersed duplication (DSD), and proximal duplication (PD), to continuously supply evolutionary material for environmental adaptation [5]. Many duplicated genes generated from duplication events lost partners and remained as singletons during evolution [6]. How plants distinguish genes produced by different mechanisms within the same content is an intriguing question. There is evidence that duplicated genes undergo different purifying selection, which is associated with gene expression, posttranscriptional regulation, and DNA methylation [7, 8]. These duplication events and the high retention rate of existing duplicated gene pairs have contributed to the abundance of duplicated genes, enhancing plant adaptations, such as disease resistance and adaptability to adversity [6, 8]. The molecular mechanisms involved in plant adaptations, such as transcriptome changes, DNA methylation, histone modification, and the m^6^A epitranscriptome, have been reviewed [3, 9–12]. However, how m^6^A modifications of multiple duplicated genes change during the formation and evolution of polyploid plants has yet to be delineated.

RNA molecules, which may be modified by a variety of chemical modifications [9], act as genetic information carriers, linking DNA to proteins and regulating multifarious biological processes [13]. N^6^-methyladensine (m^6^A), which is the most widespread modification of messenger RNA (mRNA), has been found to account for 50% of methylated nucleotides in polyadenylated mRNA in eukaryotes [14]. The dynamic and reversible m^6^A modification is regulated by writers (RNA methyltransferases, including METTL3, METTL4, WTAP, VIRMA, and HAKAI) [15–19], erasers [RNA demethylases, comprising fat-mass and obesity-associated protein (FTO) and AlkB homolog 5 (ALKBH5)] [20, 21], and readers (RNA-binding proteins that identify and combine m^6^A markers, including YTH domain family proteins) [22]. The putative m^6^A regulatory machineries were recently described in 22 plant species [9]. Loss of function of components of regulatory machineries leads to early embryonic lethality [18]. Due to advances in transcriptome-wide m^6^A sequencing and mapping technology [23], m^6^A modification has been demonstrated to have pivotal roles in biological and developmental processes of plants, such as embryo development, shoot apical meristem development, fruit ripening, enhanced resistance, and response to stresses [15, 18, 24–27]. Yu *et al*. [28] found that transgenic expression of human FTO in rice and potato mediated substantial m^6^A demethylation and increased yield and biomass by ~50%, which demonstrates the value of modulating m^6^A modifications in plant breeding. Cheng *et al*. [29] revealed that m^6^A modifications promote the biosynthesis of auxin to guarantee male meiosis and fertility in rice. Duan *et al*. [30] demonstrated that mRNA demethylation mediated by ALKBH20B affects floral transition in *Arabidopsis*. A recent study established transcriptome-wide m^6^A methylomes of 13 representative plants and revealed the conservation and diversity of m^6^A modifications in plants [31]. In paleo-polyploid maize, genes carrying m^6^A peaks in transcripts exhibit biased subgenome fractionation, which is related to multiple sequence features and asymmetric evolutionary rates [6]. However, the characteristics of m^6^A methylomes are still unknown in plant polyploidization and subsequent evolutionary processes.

As a prominent economic oil crop, *Brassica napus* L. (2*n* = 4*x* = 38, AACC) was shaped by the hybridization and subsequent WGD of *Brassica oleracea* (2*n* = 18, CC) and *Brassica rapa* (2*n* = 20, AA) ~7500 years ago [32]. These species serve as an ideal system for revealing genomic features and gene expression associated with polyploidization [11, 33]. However, the features of posttranscriptional RNA modifications in the shaping and subsequent evolution of *B. napus* are still obscure. Here, transcriptome-wide RNA m^6^A of natural *B. napus*, resynthesized *B. napus* and its parents, *B. oleracea* and *B. rapa*, were investigated. The relationship of distinct m^6^A modifications and differences in gene expression, and the distribution features of four epigenetic markers (H3K4me3, H3K27ac, H3K27me3, and DNA methylation) and m^6^A modification of different types of genes were comprehensively analyzed. Information on transcriptome-wide m^6^A modification and gene expression will provide essential resources for studying the molecular regulatory basis in *B. napus* and other polyploid plants.

## Results

### Distribution characteristics of m^6^A in four genotypes

Twenty-four m^6^A-immunoprecipitation (IP) and the corresponding non-IP control (input) libraries were constructed and sequenced, comprising natural *B. napus* (NAC), resynthesized *B. napus* (RAC), *B. oleracea* (C) and *B. rapa* (A) (Supplementary Data Table S1), with three independent biological replicates for each genotype. There were high Spearman’s correlation coefficients (not less than 0.89) among biological replicates (Supplementary Data Figs S1 and S2). The libraries contained 36–59 million raw reads (Supplementary Data Table S1), which is comparable with the sequencing depth in studies of m^6^A in tomato (20–30 million reads) [25] and strawberry (24–37 million reads) [27]. Approximately 99.99% of raw reads were clean reads after adaptor trimming and removal of low-quality reads, and 61.75%–90.44% of clean reads were mapped to the *B. napus* genome, indicating high alignment quality (Supplementary Data Table S1). We identified 5860–16 194 m^6^A peaks within 5570–14 908 gene transcripts using the MACS peak-calling algorithm in leaf tissue in four genotypes (Supplementary Data Table S1).

**Figure 1:**
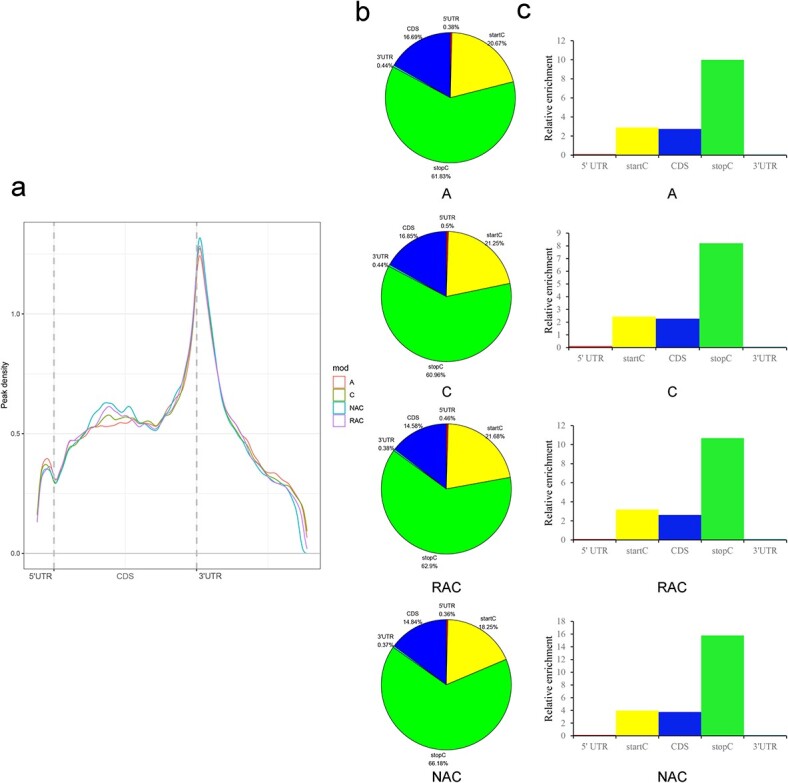
Dynamic distribution feature of m^6^A modification in leaves of A, C, RAC, and NAC. **a** Distribution of m^6^A peak along transcripts. **b** Distribution of m^6^A peaks in different transcript segments. **c** Relative enrichment of m^6^A peaks in different transcript segments.

To investigate the distribution feature of m^6^A modifications in the whole transcriptome, the transcript was divided into five contiguous segments: 3′ untranslated region (UTR), stop codon (200 bp centered on the translational stop sites, stopC), coding sequence (CDS), start codon (200 bp centered on the translational start codons, startC), and 5′ UTR. As shown in Fig. 1a, m^6^A modifications in all genotypes were highly enriched near the stopC. The pie charts show that stopC regions were enriched, exceeding 60% of the m^6^A peaks; ~20% of the m^6^A peaks were located in the startC and 14.58%–16.85% of the m^6^A peaks were positioned in the CDS of the four genotypes (Fig. 1b). The relative m^6^A enrichment analysis showed that m^6^A modifications were mainly enriched in the stopC (Fig. 1c). Thus, the transcriptome-wide mapping of m^6^A modifications showed a conserved m^6^A distribution pattern in *B. napus* and diploid progenitors.

Discriminative Regular Expression Motif Elicitation (DREME; https://meme-suite.org/meme/tools/dreme) was used to detect motifs within m6A peaks [34]. As shown in Supplemental Figure S3, the conserved RRACH (R represents A/G, A is m6A, and H represents A/C/U) consensus sequence which was detected in various organisms [6, 24-27], was also observed in B. napus and its progenitors. Surprisingly, another conserved consensus sequence URUAY (Y represents C/U), which was found in tomato [25], Arabidopsis [24, 26] and maize [6], was identified only in natural B. napus (Supplemental Figure S3). These results reflected both conservatism and diversity of m^6^A modification among these species.

To identify the relationship between gene sequence features and m^6^A modification, we performed a statistical analysis using gene sequence features (lengths of genes, exons, and introns, and number of exons) from m^6^A genes (genes carrying m^6^A peaks on transcripts) and non-m^6^A genes (genes without m^6^A on transcripts). In the four genotypes, the genes, exons, and introns were significantly longer in m^6^A genes than in non-m^6^A genes (*P* < .05) (Supplementary Data Figs S4a–c, S5a–c, S6a–c,  and S7a–c), indicating that the transcripts from longer genes were more likely to be modified with m^6^A. The same results were also observed in maize [6]. Moreover, m^6^A genes had significantly more exons than non-m^6^A genes in *Brassica* plants (*P* < .05) (Supplementary Data Figs S4d, S5d, S6d, and S7d), as seen in maize [6]. In general, these observations indicated that m^6^A modification is related to gene sequence characteristics comprising length and exon number in plants.

### Abundance of m^6^A modification and gene expression levels in the four genotypes

**Figure 2:**
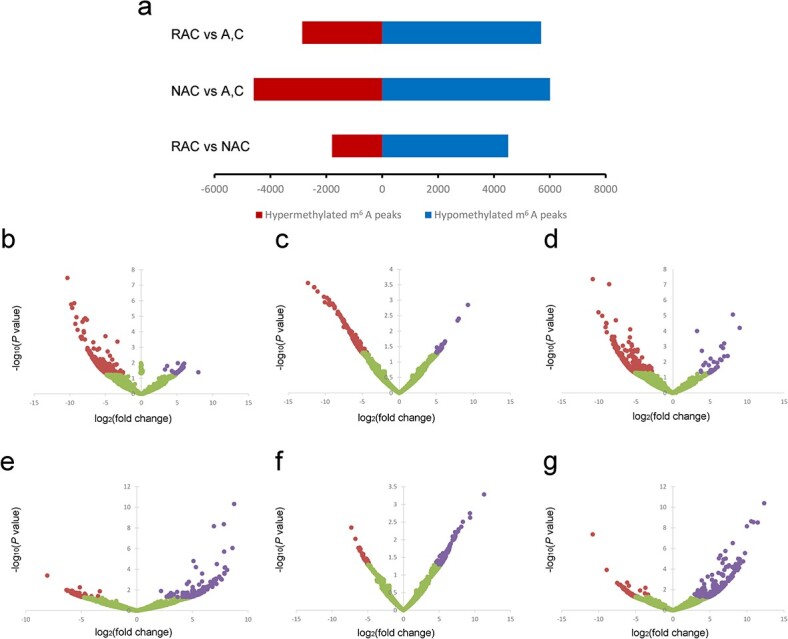
Statistics of differentially methylated peaks and relation between m^6^A modification and gene expression level in *B. napus* and diploid progenitors. **a** Differentially methylated peaks in different comparisons. **b**–**g** Volcano plots showing ratios of gene expression level of transcripts comprising (**b**) hypomethylated mRNA in RAC versus A and C, (**c**) hypomethylated mRNA in NAC versus A and C, (**d**) hypomethylated mRNA in NAC versus RAC, (**e**) hypermethylated mRNA in RAC versus A and C, (**f**) hypermethylated mRNA in NAC versus A and C, and (**g**) hypermethylated mRNA in NAC versus RAC. Red dots represent significantly downregulated mRNA, purple dots represent significantly upregulated mRNA, and green dots indicate mRNA with no significant change.

To investigate the characteristics of m^6^A modification in the shaping and subsequent evolution of *B. napus*, a differential methylation level analysis of transcripts among the genotypes was performed (Fig. 2a). Compared with its progenitors, 5688 m^6^A peaks were hypomethylated, whereas only 2864 m^6^A peaks were hypermethylated in resynthesized *B. napus* (Fig. 2b and e). Compared with resynthesized *B. napus*, 1795 hypomethylated m^6^A peaks and 4512 hypermethylated m^6^A peaks were identified in natural *B. napus* (Fig. 2d and g). Compared with diploid progenitors, a total of 6009 hypomethylated m^6^A peaks and 4956 hypermethylated m^6^A peaks were found in natural *B. napus* (Fig. 2c and f). These results indicated the remarkable differences in m^6^A methylome in the four genotypes. Gene Ontology (GO) enrichment analysis was performed to explore the potential roles of genes whose transcripts comprised differential m^6^A modifications (DMGs). DMGs between resynthesized *B. napus* and its progenitors were related to ‘macromolecule modification’, ‘cellular process’, and ‘RNA processing’, whereas DMGs between natural *B. napus* and its progenitors were involved in ‘gene expression’, ‘translation’, and ‘cellular amide metabolic process’ (Supplementary Data Table S2). In the comparison between resynthesized *B. napus* and natural *B. napus*, DMGs were highly enriched in ‘metabolic process’, ‘small molecule biosynthetic process’, and ‘cytoplasmic translation’ (Supplementary Data Table S2). Interestingly, hypomethylated mRNAs were more likely to be downregulated, whereas more hypermethylated mRNAs appeared to be upregulated (Fig. 2b–g). Notably, GO analysis of these significantly differentially expressed genes showed that they were involved in the ‘cytoplasm’.

Combined with the transcriptome results, we performed a statistical analysis of the m^6^A modification of genes expressed in the leaves of four genotypes. The percentage of m^6^A genes was significantly higher in *B. rapa* than in *B. oleracea* (Supplementary Data Fig. S8a). Although the overall proportion of the m^6^A gene in resynthesized *B. napus* did not change significantly compared with progenitors, the difference between the subgenomes A_n_ and C_n_ diminished. On the other hand, the proportion of the m^6^A genes in each subgenome of natural *B. napus* was notably increased. These results suggested that hybridization and WGD reduced the difference in the proportion of m^6^A genes between subgenomes of resynthesized *B. napus*. Compared with resynthesized *B. napus*, m^6^A genes in both subgenomes was increased in natural *B. napus*. To explore the potential relationship between m^6^A modification and gene expression levels, the mRNA abundance of gene transcripts exhibiting differential m^6^A modification was compared. Compared with non-m^6^A genes, m^6^A genes showed significantly lower gene expression levels in all genotypes, indicating that the lower mRNA abundance of transcripts may be related to m^6^A modifications (Supplementary Data Fig. S8b).

We then compared the evolutionary rates (*ω*), calculated by dividing non-synonymous substitution (*K*_a_) by synonymous sites (*K*_s_), of m^6^A genes and non-m^6^A genes of *B. napus* and diploid progenitors (Supplementary Data Table S3). The evolutionary rates were analyzed employing interspecific putatively orthologous sequences between *B. napus* and *Arabidopsis*. The m^6^A genes had notably higher *ω* values than non-m^6^A genes in progenitors, which is consistent with diploid maize [6]. However, there was no significant difference in the *ω* value between the two categories of genes in resynthesized *B. napus*. Surprisingly, there was a reversal in the natural *B. napus*: the non-m^6^A genes had notably higher *ω* values than m^6^A genes. These results indicated that the m^6^A genes had higher evolutionary rates than non-m^6^A genes in progenitors, but they were neutralized after hybridization and polyploidization and reversed during subsequent evolution.

To explore the differences in m^6^A modification in the early shaping and subsequent evolution of *B. napus*, we divided genes into four patterns (Table 1). A gene whose transcript was modified by m^6^A in both genotypes in a comparison (e.g. resynthesized *B. napus* versus progenitors) was designated as pattern I, and a gene whose transcript was not modified by m^6^A in both genotypes was designated as pattern II. A gene whose transcript was modified in one genotype but not in another genotype was designated as pattern III, and the modified pattern in reverse was designated as pattern IV. We found no differences in m^6^A patterns in >75% of gene transcripts in the three comparisons. Compared with progenitors, there was little difference in the proportion of patterns III and IV in resynthesized *B. napus*. Compared with natural *B. napus*, the proportion of transcripts in pattern IV was significantly higher than that in pattern III, which was consistent with the previously described observation of increased m^6^A genes of natural *B. napus* (Supplementary Data Fig. S8a). These observations indicated that most mRNA m^6^A modification patterns were maintained in the shaping and evolution of *B. napus*.

**Table 1:** Statistics of transcripts in four patterns of m^6^A modification differences.

Pattern	m^6^A Modification differences	Numbers of transcripts (%)		
		A, C versus RAC	A, C versus NAC	RAC versus NAC
I	m^6^A → m^6^A	8397 (35.9)	9094 (39.1)	9319 (43.1)
II	non-m^6^A → non-m^6^A	11 123 (47.5)	8562 (36.8)	7655 (35.4)
III	m^6^A → non-m^6^A	2002 (8.6)	1398 (6)	703 (3.3)
IV	non-m^6^A → m^6^A	1875 (8)	4193 (18)	3939 (18.2)

**Figure 3:**
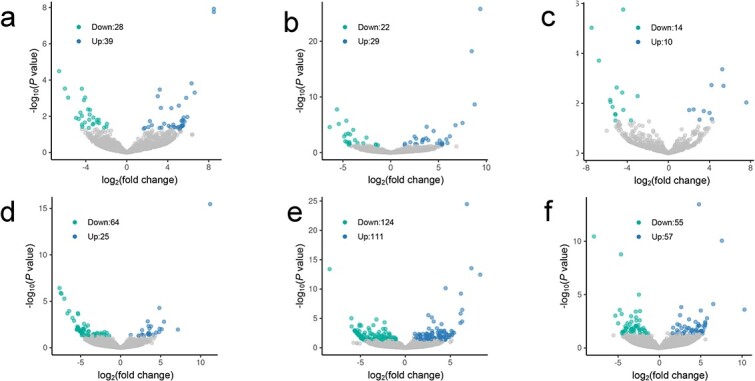
Gene expression level analysis of transcripts in patterns III and IV. **a** Transcripts in pattern III of A and C versus RAC. **b** Transcripts in pattern III of A and C versus NAC. **c** Transcripts in pattern III of NAC versus RAC. **d** Transcripts in pattern IV of A and C versus RAC. **e** Transcripts in pattern IV of A and C versus NAC. (f) Transcripts in pattern IV of NAC versus RAC. Green dots represent significantly downregulated mRNA, blue dots represent significantly upregulated mRNA, and gray dots represent mRNA with no significant change.

To explore the relationship of the presence or absence of m^6^A modification with mRNA abundance, we analyzed the mRNA abundance of gene transcripts in patterns III and IV among genotypes. More transcripts with m^6^A modification in progenitors but free of m^6^A modification in resynthesized *B. napus* were upregulated*,* whereas more transcripts without m^6^A modification in progenitors but with m^6^A modification in resynthesized *B. napus* were downregulated (Fig. 3a and d). These results reflected the negative relationship of m^6^A modification and gene expression level. Similar observations were also found in the comparison between natural *B. napus* and diploid progenitors (Fig. 3b and e). However, more transcripts with reversed m^6^A modification between resynthesized and natural *B. napus* were downregulated (Fig. 3c and f).

### Distribution characteristics of epigenetic modifications around genes of m^6^A peaks in four genotypes

**Figure 4:**
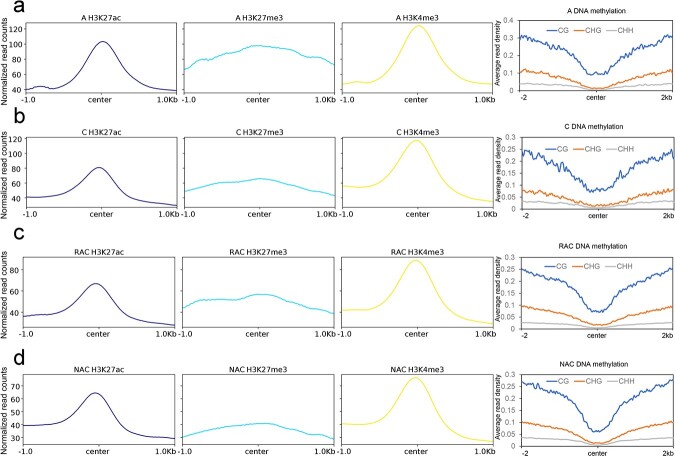
Association between four epigenetic modifications and m^6^A modification. **a**–**d** Distribution of H3K27ac, H3K27me3, H3K4me3, and DNA methylation around m^6^A peaks in A *B. rapa* (**a**), C *B. oleracea* (**b**), RAC resynthesized *B. napus* (**c**), and NAC natural *B. napus* (**d**). Center, m^6^A peak center.

Previous studies revealed that histone modification (H3K36me3) guides m^6^A RNA modification deposition co-transcriptionally in human and mouse, and showed an intimate linkage between H3K36me2 modification and m^6^A modification in *Arabidopsis* [35, 36]. To explore the distribution features of epigenetic modifications around genes of m^6^A peaks in *B. napus*, we investigated the distribution of histone modifications (H3K4me3, H3K27ac, and H3K27me3) and DNA methylation around genes of the m^6^A peaks. We found that active histone modifications (H3K27ac and H3K4me3) were enriched around genes of the m^6^A peak center, whereas the repressive histone modification H3K27me3 was only slightly enriched around genes of the m^6^A peak center compared with both flanks in all genotypes (Fig. 4), which reflected the co-occurrence of H3K27ac and H3K4me3 modifications and m^6^A modification. Moreover, CG, CHG, and CHH DNA methylation was depleted around genes of the m^6^A peak center, and DNA methylation levels increased gradually with the distance from genes of the m^6^A peak center (Fig. 4). These results demonstrate that genes of the m^6^A peaks were enriched in active histone modifications (H3K27ac and H3K4me3) but depleted in DNA methylation in *B. napus* and diploid progenitors.

### Singletons exhibited distinct distribution features of m^6^A modification compared with duplicated genes

**Figure 5:**
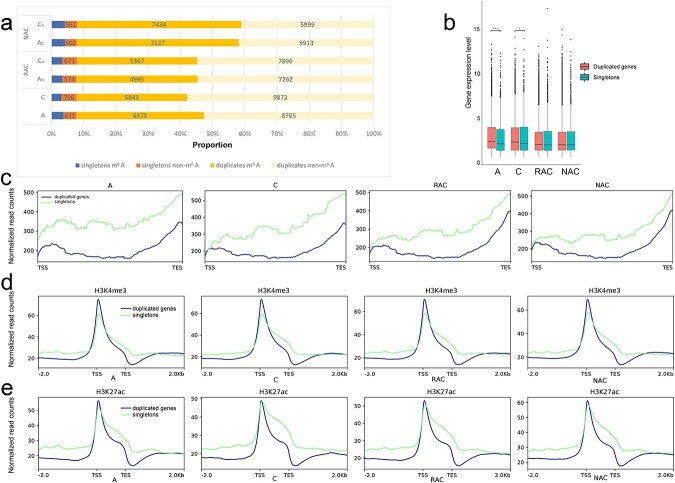
Proportion, gene expression level, metagenomic profiles of singletons and duplicated genes. **a** Proportion of different m^6^A gene modified singletons and duplicated genes. **b** Gene expression level of singletons and duplicated genes. **c** Metagenomic profiles of m^6^A modification of transcripts of singletons and duplicated genes. **d** Metagenomic profiles of H3K4me3 modification of singletons and duplicated genes. **e** Metagenomic profiles of H3K27ac modification of singletons and duplicated genes. The Wilcoxon rank sum test was performed for statistical analysis. ^*^P < .05, ^***^P < .001.

A previous study revealed that genome duplication is a propensity of *Brassica*, in which diploid *B. rapa* and *B. oleracea* experienced an aggregate 36× multiplication (3 × 2 × 2 × 3) and allopolyploid *B. napus* experienced an aggregate 72× multiplication (3 × 2 × 2 × 3 × 2) [32]. A member of the duplicated gene pair produced by WGD may be lost (fractionated) during evolution, the remaining one being known as a singleton. To explore the distribution of m^6^A modification of singletons and duplicated genes, a statistical analysis of the duplication status of the existing genes was performed. The proportions of singletons/duplicated genes of each genome/subgenome were relatively small (8.4%–8.7%) (Fig. 5a), indicating that most of the genes in each genome/subgenome still existed as duplicated genes, and there was no obvious biased gene fractionation among genomes/subgenomes. These results are different from those observed in maize, in which the proportion of singletons was higher than that of duplicated genes, and there was a significant bias in gene fractionation between the two subgenomes (maize1 > maize2) [6]. These differences may be due to the higher frequency of WGD events, and the timing of the last WGD was much more recent in *B. napus* than in maize [6, 32]. The proportion of the m^6^A gene of singletons was notably greater than that of duplicated genes in *B. rapa* and subgenome A_n_ of resynthesized *B. napus*, but not in subgenome A_n_ of natural *B. napus* (Fig. 5a). There was no significant difference between the proportion of the m^6^A gene of singletons and duplicated genes of *B. oleracea* and subgenome C_n_ of resynthesized *B. napus*, but the proportion of the m^6^A gene of duplicated genes of subgenome C_n_ of natural *B. napus* was significantly higher (Fig. 5a). These results indicated that the m^6^A modification of singletons and duplicated genes was different between the two diploid progenitors, and this difference was inherited after hybridization and WGD, but changed in the subsequent evolution process.

To compare the features of m^6^A modifications and gene expression levels of singletons and duplicated genes, we analyzed m^6^A modifications and mRNA abundance among these transcripts. Although the m^6^A modification of transcripts of both singletons and duplicated genes was highly enriched around the transcript start site (TSS), the transcripts of singletons exhibited higher m^6^A modification than the duplicated genes in all genotypes (Fig. 5c). Moreover, compared with progenitors, a smaller difference in m^6^A modification around the transcript end site (TES) and TSS between singletons and duplicated genes was found in *B. napus*. Interestingly, the gene expression of singletons was significantly lower than that of duplicated genes in progenitors, but there was no obvious difference in *B. napus* (Fig. 5b). Then, we analyzed three histone modifications and DNA methylation among singletons and duplicated genes of four genotypes. As shown in Fig. 5d and e and Supplementary Data Fig. S9a, the repressive histone marker H3K27me3 was primarily distributed along the gene body between the TSS and TES, while active histone markers (H3K4me3 and H3K27ac) were primarily located around the TSS. The singletons showed higher active histone markers except around the TSS (Fig. 5d and e), and higher repressive histone markers (H3K27me3) (Supplementary Data Fig. S9a) and DNA methylation (Supplementary Data Fig. S9b–d). These results reflected distinct distribution features of DNA methylation, H3K4me3, H3K27ac, and H3K27me3 modifications, and m^6^A modification of singletons and duplicated genes.

We compared the evolutionary rates of singletons and duplicated genes of *B. napus* and diploid progenitors. The singletons had significantly higher *ω* values than duplicated genes in all genotypes (Supplementary Data Fig. S10). As shown in Table 2, regardless of whether the transcripts of genes were modified by m^6^A, singletons had significantly higher *ω* values than duplicated genes in the genome/subgenomes in all genotypes, indicating that singletons have experienced stronger purifying selection than duplicated genes. These results suggested that singletons whose transcripts showed higher m^6^A modification have experienced faster sequence divergence than duplicated genes. The duplicated genes of genome/subgenome C had significantly higher *ω* values than those of genome/subgenome A of *B. napus*, but there was no obvious difference between singletons of the two genomes/subgenomes. These results indicated that the duplicated genes of genome/subgenome C have experienced stronger purifying selection than those in genome/subgenome A.

**Table 2:** Evolutionary rates (*ω*) of m^6^A, non-m^6^A singletons, and duplicated genes in genomes/subgenomes.

Genotype	Genome/subgenome	Evolutionary rates (*ω*)		*P* value	Evolutionary rate (*ω*)		*P* value
		m^6^A Singletons	m^6^A Duplicated genes		Non-m^6^A singletons	Non-m^6^A duplicated genes	
A, C	A	0.1813 ± 0.1078	0.1493 ± 0.0944	<0.001	0.1817 ± 0.0956	0.1443 ± 0.0974	<0.001
	C	0.1839 ± 0.1089	0.1549 ± 0.1010	<0.001	0.1855 ± 0.0994	0.1499 ± 0.1043	<0.001
	*P* value	0.7353	0.008577		0.704	0.001649	
RAC	A_n_	0.1743 ± 0.1097	0.1414 ± 0.0928	<0.001	0.1845 ± 0.0951	0.1437 ± 0.0973	<0.001
	C_n_	0.1769 ± 0.1047	0.1467 ± 0.0993	<0.001	0.1880 ± 0.0997	0.1496 ± 0.1038	<0.001
	*P* value	0.6922	0.01415		0.78	0.001028	
NAC	A_n_	0.1757 ± 0.1069	0.1374 ± 0.0928	<0.001	0.1820 ± 0.0916	0.1496 ± 0.0990	<0.001
	C_n_	0.1760 ± 0.0989	0.1424 ± 0.0983	<0.001	0.1982 ± 0.1077	0.1549 ± 0.1061	<0.001
	*P* value	0.7983	0.007345		0.1714	0.01457	

A, *B. rapa*; C, *B. oleracea*; RAC, resynthesized *B. napus*; NAC, natural *B. napus*.

### Distribution features of m^6^A modification in five types of duplicated genes

**Figure 6:**
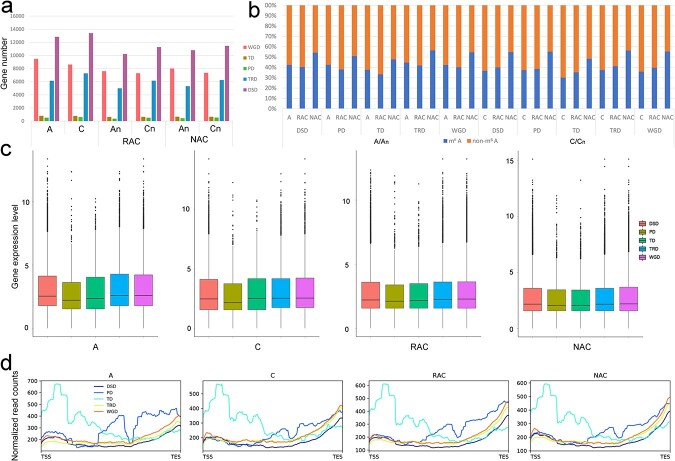
Number, gene expression level, and m^6^A modification distribution of five types of duplicated gene. **a** Number of five kinds of duplicated genes in *B. napus* and diploid progenitors. **b** Proportion of m^6^A genes in five kinds of duplicated genes in each genome/subgenome. **c** Gene expression level of five types of duplicated genes in *B. napus* and diploid progenitors. **d** Metagenomic profiles of m^6^A peak distribution of five types of duplicated genes in *B. napus* and diploid progenitors.

Given the high proportion of duplicated genes due to the complex WGD events both in *B. napus* and in diploid progenitors, we divided the duplicated genes identified in our study into five types for in-depth analysis according to a previous study [5]. The number of genes from WGD, DSD, and TRD identified in this study was higher than that from TD and PD in all genotypes (Fig. 6a). Then, we counted the proportion of the m^6^A gene of each type of duplicated gene in each genotype and found that the pattern of change was different between the two subgenomes (Fig. 6b). In resynthesized *B. napus*, the proportion of the m^6^A gene in subgenome A_n_ decreased, but the proportion of m^6^A gene in subgenome C_n_ increased compared with progenitors. The proportion of the m^6^A gene in both subgenomes increased in natural *B. napus* compared with resynthesized *B. napus*. These results indicated that hybridization and WGD balanced the proportion of the m^6^A gene between subgenomes of *B. napus*, which was reflected in all kinds of duplicated genes (Fig. 6b; Supplementary Data Fig. S8a).

We analyzed m^6^A modifications among five types of duplicated genes and found that transcripts of TD-derived genes exhibited a different distribution of m^6^A modification, which was highly enriched around the TSS (Fig. 6d). In contrast, m^6^A modification was highly enriched around the TES in the gene transcripts of other duplicated genes around the TES. Around the TES, the transcripts from WGD-derived genes exhibited the highest level of m^6^A modification, followed by transcripts from TRD-, DSD- and TD-derived genes. Interestingly, only transcripts of PD- and TD-derived genes were highly enriched in m^6^A modification of the body of gene transcripts. Then, we analyzed the three histone modifications and DNA methylation among the five types of duplicated genes and found that the WGD-derived genes exhibited the highest level of active histone markers (H3K4me3 and H3K27ac) (Supplementary Data Fig. 11a and b) but the lowest level of DNA methylation (Supplementary Data Fig. 12a–c), which was consistent with their highest gene expression level (Fig. 6c). The WGD-, TRD- and DSD-derived genes showed opposite distribution trends in the histone markers and DNA methylation: WGD-derived genes had the highest level of histone markers but lowest DNA methylation, while DSD-derived genes had the highest level of DNA methylation but the lowest level of histone markers (Supplementary Data Figs S11 and S12). Surprisingly, the PD-derived genes exhibited the highest level of repressive histone markers (H3K27me3) and DNA methylation in the CG content but the lowest level of active histone markers. Interestingly, active histone markers of the PD- and TD-derived genes showed opposite trends in the gene bodies of progenitors, but there was little difference in *B. napus* (Supplementary Data Fig. S11a and b). These results indicated that genes derived from various duplication events showed distinct distribution features of these epigenetic modifications and m^6^A modification.

**Figure 7:**
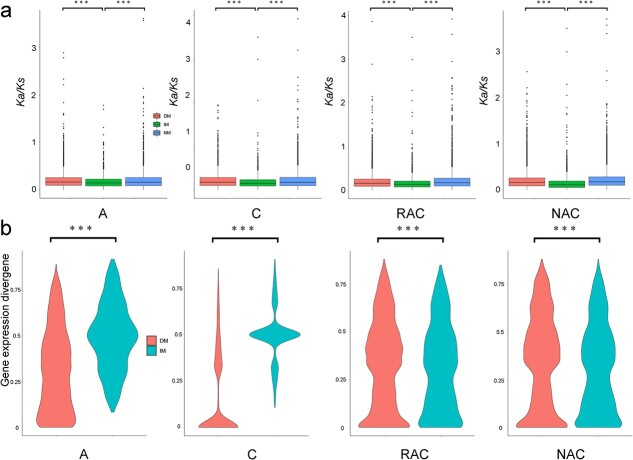
*K*_a_/*K*_s_ of duplicated gene pairs with different m^6^A modification patterns and gene expression divergence of duplicated genes in IM and DM. **a***K*_a_/*K_s_* of duplicated gene pairs with different m^6^A modification patterns on the whole. **b** Gene expression divergence of duplicated genes in IM and DM. IM, identical m^6^A pattern; DM, diverged m^6^A pattern; NM, non-m^6^A pattern.

We compared the evolutionary rates of five types of duplicated genes. The evolutionary rates (*ω*) were analyzed using putatively orthologous sequences within *B. napus*. We found that TD- and PD-derived genes had higher evolutionary rates than WGD-, TRD-, and DSD-derived genes (Supplementary Data Fig. S13a). To investigate the association between m^6^A modification and evolutionary rates of genes, we analyzed the *ω* values of duplicated gene pairs with distinct m^6^A modification patterns: the identical m^6^A (IM) pattern (both gene transcripts of partners had m^6^A peaks), diverged m^6^A (DM) pattern (only a gene transcript of partners carrying m^6^A peaks), and non-m^6^A (NM) pattern (neither gene transcript of partners had m^6^A peaks). As shown in Fig. 7a, the *ω* values of IM were significantly lower than those of DM and NM in all genotypes, which indicated that the transcripts of conserved duplicated gene pairs are more likely to carry m^6^A peaks. Comparing the *ω* values of the three patterns in the five types of duplicated genes (Supplementary Data Fig. S13b), only the TD- and PD-derived genes in *B. rapa* did not follow this rule.

We investigated the association between m^6^A modification and expression divergence (ED) of duplicated genes. The ED was calculated following the formula used in a previous study with minor modification: ED = |(E1 − E2)|/(E1 + E2), where E1 and E2 represent the gene expression levels of two genes in a duplicated gene pair [6]. The ED of the IM pattern was significantly higher than that of the DM pattern in progenitors, but the opposite was observed in *B. napus*, which indicated that hybridization and WGD changed the gene divergence of duplicated genes with different m^6^A modification patterns (Fig. 7b). We found that the methylated partners of the DM pattern exhibited a lower mRNA abundance than the non-methylated partners (Supplementary Data Fig. S14). These observations indicated that m^6^A modification was negatively associated with mRNA abundance, but hybridization and WGD may change the connection between m^6^A modification and the expression divergence of duplicated genes.

### Dosage-dependent and dosage-independent genes displayed different distributions of m^6^A modification and four epigenomic markers

**Figure 8:**
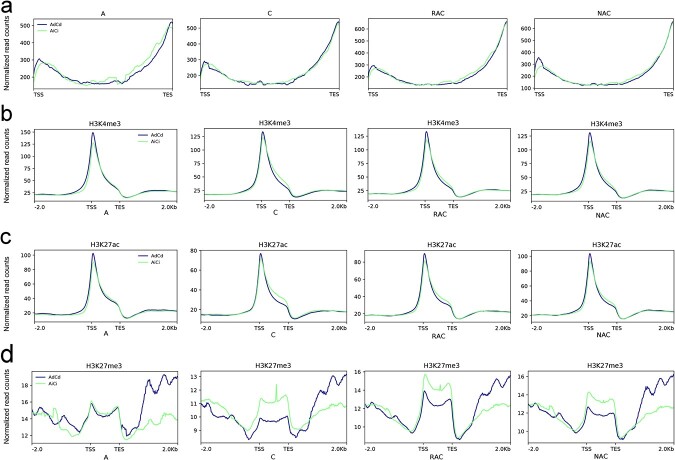
Distribution of m^6^A modifications and three histone modifications of dosage-dependent and independent genes in *B. napus* and diploid progenitors. **a** Distribution of m^6^A modifications. **b** Distribution of H3K4me3 modifications. **c** Distribution of H3K27ac modifications. **d** Distribution of H3K27me3 modifications.

As an important evolutionary mechanism, gene dosage balance guarantees normal expression of duplicated genes. Altered gene dosage may lead to gene expression changes. Therefore, the expression and fate of duplicated genes are influenced by the evolutionary mechanism of gene dosage balance [37]. Tan *et al*. [37] divided genes of *B. napus* into two categories according to the coefficients of determination (*R*^2^): dosage-dependent (*R*^2^ > .59) genes in subgenome A_n_ (Ad) and in subgenome C_n_ (Cd) and dosage-independent (*R*^2^ < .59) genes in subgenome A_n_ (Ai) and in subgenome C_n_ (Ci). We explored the distribution of m^6^A modification and four epigenetic markers of these two kinds of genes in four genotypes. The AdCd genes had higher levels of m^6^A modification around the TSS and TES (Fig. 8a), active histone markers around the TSS (H3K4me3 and H3K27ac) (Fig. 8b and c), DNA methylation of CG content in the gene body (Supplementary Data Fig. S15a), and repressive histone markers downstream of the TES (H3K27me3) (Fig. 8d), whereas AiCi genes had higher repressive histone markers in the gene body (Fig. 8d), and DNA methylation of CHG and CHH content in the gene body (Supplementary Data Fig. S15b and c). These results suggested that the dosage-dependent and dosage-independent genes showed different distribution features of three histone markers, DNA methylation in genes, and m^6^A modifications in transcripts, which may influence the differentiation of dosage-related duplicated genes in *B. napus* and its diploid progenitors.

## Discussion

### Conservation and variation of the distribution of m^6^A modification

As the most prevalent modification in transcripts, m^6^A modification has been demonstrated to play an essential role in developmental and biological processes [38]. Depending on the m^6^A distribution and the sequence contexts within transcripts, m^6^A modification can promote the degradation of mRNAs or stabilize mRNAs [27]. Recent studies on m^6^A have focused on biological development and the response to adversity [15, 26]. However, the characteristics of m^6^A modification during the early formation and subsequent evolution of polyploid plants is still unclear. Therefore, transcriptome-wide m^6^A modifications were comprehensively analyzed using allopolyploid *B. napus* and diploid progenitors as an ideal system. Overall, m^6^A modification was notably enriched around the stopC region and in the 3′ UTR in *B. napus* and diploid progenitors (Fig. 1a), similar to the distribution observed in *Arabidopsis* [26, 39], tomato [25], strawberry [27], maize [6], human, and mouse [35]. These observations reflected that the distribution of m^6^A modification was highly conserved in eukaryotes, including both plant and animal kingdoms. Additionally, there was a minor m^6^A modification enrichment around the 5′ UTR near the startC in *B. napus* and its progenitors (Fig. 1a). A similar summit of m^6^A peaks was also found in *Arabidopsis* [26, 39], strawberry [27], and rice [40] but not in tomato [25], maize [6], human [35], or mouse [23, 35]. The diverse distribution among these species may be related to species-specific genomic organizations and m^6^A regulatory mechanisms, which reflect the diversity of m^6^A methylomes. More interestingly, we observed that species with higher m^6^A modification enrichment around the stop codon and 3′ UTR also had higher m^6^A modification in the CDS but lower m^6^A modification within the 5′ UTR in *B. napus* and diploid progenitors. The opposite trend of m^6^A modification enrichment has also been observed between the region around the startC and stopC of strawberry in the ripening process [27], *Arabidopsis* under different concentrations of salt stress [26], and in callus and leaf of rice [40]. These phenomena suggest that there may be a conserved antagonistic m^6^A modification mechanism regulating m^6^A enrichment at both ends of the transcripts in these species.

### m^6^A modification showed a multifaceted relationship with gene expression level

As a reversible internal RNA modification in mRNA, m^6^A modifications regulate gene expression as key posttranscriptional regulators [38]. In mammals, the m^6^A modification level is generally negatively associated with the gene expression level [16]. During tomato ripening, differential m^6^A modification was found mainly near the stopC regions or in the 3′ UTR, which was generally negatively correlated with the gene expression level [25]. In the callus and leaves of rice, the enrichment abundance of m^6^A peaks was also negatively correlated with the gene expression level [40]. These observations suggested that m^6^A modification was negatively correlated with the gene expression level overall. However, the overall positive relationship between m^6^A modification and gene expression levels during tomato expansion was revealed by Zhou *et al*. [25]. Here, we found a complex relationship between the abundance of m^6^A modifications and gene expression level. The differential methylation level analysis and differential gene expression analysis of all transcripts showed that the enrichment abundance of m^6^A modification was positively correlated with gene expression overall (Fig. 2b–g). However, the presence of m^6^A in transcripts originally free of m^6^A modification was more likely to decrease gene expression (Fig. 3d–f). The absence of m^6^A transcripts tended to upregulate gene expression (Fig. 3a–c). Methylated partners of duplicated genes exhibited lower gene expression levels than the non-methylated partners (Supplementary Data Fig. S14). These observations reflect the opposite roles in the gene expression level of the presence/absence and the differential degree of enrichment of m^6^A modification in transcripts. In a study of strawberry ripening, m^6^A modification was found to have diverse regulatory roles on mRNAs depending on the distribution of m^6^A [27]. The m^6^A modifications located in the 3′ UTR or around stopC regions tended to be negatively correlated with gene expression, whereas those in CDS regions were more likely to stabilize the mRNAs [27]. These findings suggest that m^6^A may play unique roles in mRNAs, possibly due to its degree of enrichment, presence or absence, and different localization.

### Duplicated genes produced by various mechanisms exhibited distinct distributions of m^6^A modification

In plants, gene duplication events, including WGD events and single-gene duplication events, supply many evolutionary raw materials to adapt to changing conditions [41]. To adapt to rapidly changing environments, plants underwent a higher frequency of TD than WGD [42]. TD-derived genes were more likely to be related to stress-associated functions than others [43]. Therefore, the expansion of stress-related genes by TD in plants is considered to be a protective mechanism against harmful stresses [43]. Some ancient TD-derived genes were interrupted by other genes to form proximal duplicates [44]. Plants may need to constantly acquire new TDs and PDs to adapt to drastic environmental changes [5, 42]. In this study, we found that frequent WGDs resulted in a high proportion of duplicated genes in *B. napus* and diploid progenitors (Fig. 5a). A previous study suggested that gene expression divergence between duplicated genes might make *B. napus* more flexible in responding to abiotic stress [45]. Our study suggested that there was a great difference in gene expression divergence between IM and DM duplicated genes (Fig. 7b). Hybridization and WGD reduced the difference in the proportion of m^6^A genes of five types of duplicated genes between the two subgenomes of *B. napus* (Fig. 6b). We wondered whether different types of genes derived from various duplication events faced the same regulation in the same genetic regulatory environment. The transcripts of singletons exhibited higher m^6^A modification than that of the duplicated genes on the whole in *B. napus* and diploid progenitors (Fig. 5c). When duplicated genes were divided into five types, we found that transcripts from these genes exhibited different distributions of m^6^A modification (Fig. 6d). Only TD-derived genes have higher enrichment of m^6^A modifications around TSS than TES in transcripts. Transcripts of TD- and PD- derived genes have a minor enrichment of m^6^A modifications in the gene body. Transcripts of WGD-derived genes showed higher m^6^A modifications than DSD- and TRD-derived genes in the four genotypes (Fig. 6d). A previous study revealed that H3K36me3 guides m^6^A deposition globally in animals [35]. Shim *et al*. [36] revealed a high correlation between H3K36me2 and m^6^A modification in plants. In this study, we found that the singletons had obviously higher repressive histone markers (H3K27me3) and DNA methylation not only in the gene body but also in the upstream and downstream regions of the gene (Supplementary Data Fig. S9a), and displayed overall higher enrichment of m^6^A modifications in the transcripts. Interestingly, in contrast to the distribution of m^6^A modification, the singletons and the duplicated genes have more active histone markers (H3K4me3 and H3K27ac) (Fig. 5d and e) around the TSS than the TES. The PD-derived genes showed lower levels of active histone markers (H3K4me3 and H3K27ac) (Supplementary Data Fig. S11a and b), higher levels of repressive histone markers (H3K27me3) (Supplementary Data Fig. S11c), and higher levels of DNA methylation in the gene body (Supplementary Data Fig. S12a–c), and displayed unique m^6^A modifications between the TSS and TES (Fig. 6d). The TD-derived genes exhibited lower levels of active histone markers and repressive histone markers and showed additional higher enrichment of m^6^A modifications around the TSS (Fig. 6d, Supplementary Data Fig. S11). These observations demonstrated that different types of duplicated genes exhibited distinct distributions of epigenetic modifications and m^6^A modifications in transcripts, and the specific distribution patterns of m^6^A modification may be an important marker for distinguishing the transcripts of different types of genes. Additionally, we found that TDs and PDs have some common features compared with other types of duplicated genes, such as lower active histone markers (Supplementary Data Fig. S11a and b), unique m^6^A modification in the middle of transcripts (Fig. 6d), higher evolutionary rates (Supplementary Data Fig. S13a), and lower gene expression levels (Fig. 6c). These findings suggest that TDs and PDs experience faster functional divergence, while epigenetic modifications (H3K4me3, H3K27ac, H3K27me3, and DNA methylation) and m^6^A modifications appear to play a major part in this process.

## Materials and methods

### Plant materials

Seeds of natural *B. napus* L. (cv. ‘Darmor’), resynthesized *B. napus* L. (HC-2) and its progenitors, *B. rapa* L. (cv. ‘9JC002’) and *B. oleracea* L. (cv. ‘3YS013’) were provided by the Oil Crop Research Institute, Chinese Academy of Agricultural Sciences, China. All plants were cultivated in light incubators at 23°C with a day/night photoperiod of 16 hours/8 hours. Leaves of 1-month-old seedlings of four genotypes were harvested in triplicate, frozen promptly in liquid nitrogen, and then preserved at −80°C.

### RNA-sequencing and data analysis

Total RNA of all samples was extracted by using a TRIzol Kit (Promega, Madison, USA). Then, the rRNAs were eliminated using a NEBNext rRNA Depletion Kit (New England Biolabs, MA, USA) following the manufacturer’s instructions. RNA libraries were constructed with the NEBNext^®^ Ultra II Directional RNA Library Prep Kit (New England Biolabs, MA, USA) according to the manufacturer’s instructions. The quality and quantity of RNA\ libraries was controlled by the library BioAnalyzer 2100 system (Agilent Technologies, USA). An Illumina Novaseq 6000 instrument was used to perform library sequencing with 150 bp paired end reads and Q30 was used to control quality. After removing 3′ adaptor-trimmed and low-quality reads by Cutadapt software (v1.9.3) [46], the high-quality clean reads were aligned to the reference *B. napus* genome v5 (http://www.genoscope.cns.fr/brassicanapus/data/) with HISAT2 software (v2.0.4) [47]. HTSeq software (v0.9.1) was used to get raw counts, and then normalization of gene expression levels in terms of FPKM (fragments per kilobase million) was performed by edgeR. Differentially expressed genes were detected according to the criteria of *P*-value ≤.05 and |log_2_fold change| ≥1 by edgeR. g:Profier (https://biit.cs.ut.ee/gprofiler/gost) was used to perform GO enrichment analysis [48].

### Methylated RNA immunoprecipitation with next-generation sequencing and data analysis

Methylated RNA immunoprecipitation with next-generation sequencing (MeRIP-seq) was carried out based on the method reported by a previous study with slight modifications [23]. In brief, fragmented RNA was incubated with anti-m^6^A polyclonal antibody (Synaptic Systems, 202 003) in IP buffer for 2 hours at 4°C. The mixture was incubated with protein-A beads (Thermo Fisher, Waltham, MA, USA) at 4°C for 2 hours for IP. The bound RNA was eluted from the beads in IP buffer using *N*^6^-methyladenosine (Berry & Associates, PR3732) and then extracted with Trizol reagent (Thermo Fisher, Waltham, MA, USA) based on the manufacturer’s instructions. An RNA-seq library was constructed using purified RNA with the NEBNext^®^ Ultra II Directional RNA Library Prep Kit (NEB). An Illumina NovaSeq 6000 instrument was used to conduct library sequencing with 150 bp paired-end reads and Q30 was used for control quality. After removing 3′ adaptors and low-quality reads with Cutadapt software (v1.9.3), clean reads were aligned to the reference *B. napus* genome using HISAT2 software. MACS software was used to identify methylated sites on RNAs (peaks) with the following parameters: -p 0.05 -f BED —tsize 150 —keep-dup = all —verbose 3 —nomodel [49]. Differentially methylated sites were detected by diffReps with a criterion of fold change in m^6^A enrichment ≥2 and *P* < .0001 [50]. m^6^A motifs were identified by DREME with a limited length of six nucleotides.

### Data analysis of four epigenetic markers

Data on histone modifications (H3K4me3, H3K27me3, and H3K27ac) and DNA methylation were obtained from a previous study [11]. Data on histone modifications were analyzed using deepTools [51], while data on DNA methylation were analyzed using BatMeth2 [52].

### Evolutionary rate analysis of orthologous and paralogous genes

The evolutionary rates (*ω*) were calculated as *K*_a_/*K*_s_. *K*_a_ and *K*_s_ were calculated using PAML 4.8 [53]. The *ω*, *K*_a_, and *K*_s_ values of the singletons and duplicated genes in *B. napus* were calculated by comparing putatively orthologous sequences between *B. napus* and *Arabidopsis thaliana*, and those of duplicated gene pairs were estimated by comparing paralogous sequences between gene pairs in *B. napus*.

### Statistical analysis

The Wilcoxon rank sum test was carried out using the wilcox.test function and the χ^2^ test was accomplished using the chisq.test function in the R package.

## Acknowledgements

We thank Dahu Zou and Yi Li at Wuhan University and Guoliang Li at Huazhong Agricultural University for their valuable advice on data analysis. This work was supported by the National Natural Science Foundation of China (31970241).

## Author contributions

This study was designed by J.W. and Z.L. Z.L. analyzed the data and wrote the manuscript. X.W. provided the experimental materials. M.L. was responsible for planting materials. M.L. provided data on histone modifications and DNA methylation. The manuscript was revised by J.W. and X.W. All authors reviewed and approved the manuscript.

## Data availability

MeRIP-seq and RNA-seq data in this research have been stored in the National Center for Biotechnology Information (NCBI) Sequence Read Archive (SRA) with the accession numbers SRR16842417–SRR16842428 and SRR16842657–SRR16842668. The data on four epigenetic markers can be obtained from NCBI with the accession codes SRR13306925–SRR13306936 (WGBS) and SRR13318007–SRR13318030 (ChIP-seq).

## Conflict of interest

The authors declare that they have no conflict of interest.

## Supplementary data

Supplementary data is available at *Horticulture Research* online.

## Supplementary Material

Web_Material_uhac230
