# Supplementary figures and images for: The characteristics of mRNA m6A methylomes in allopolyploid Brassica napus and its diploid progenitors
Source: Hortic Res. 2022 Oct 11;10(1):uhac230. doi: 10.1093/hr/uhac230 (PMC9832873; doi:10.1093/hr/uhac230)

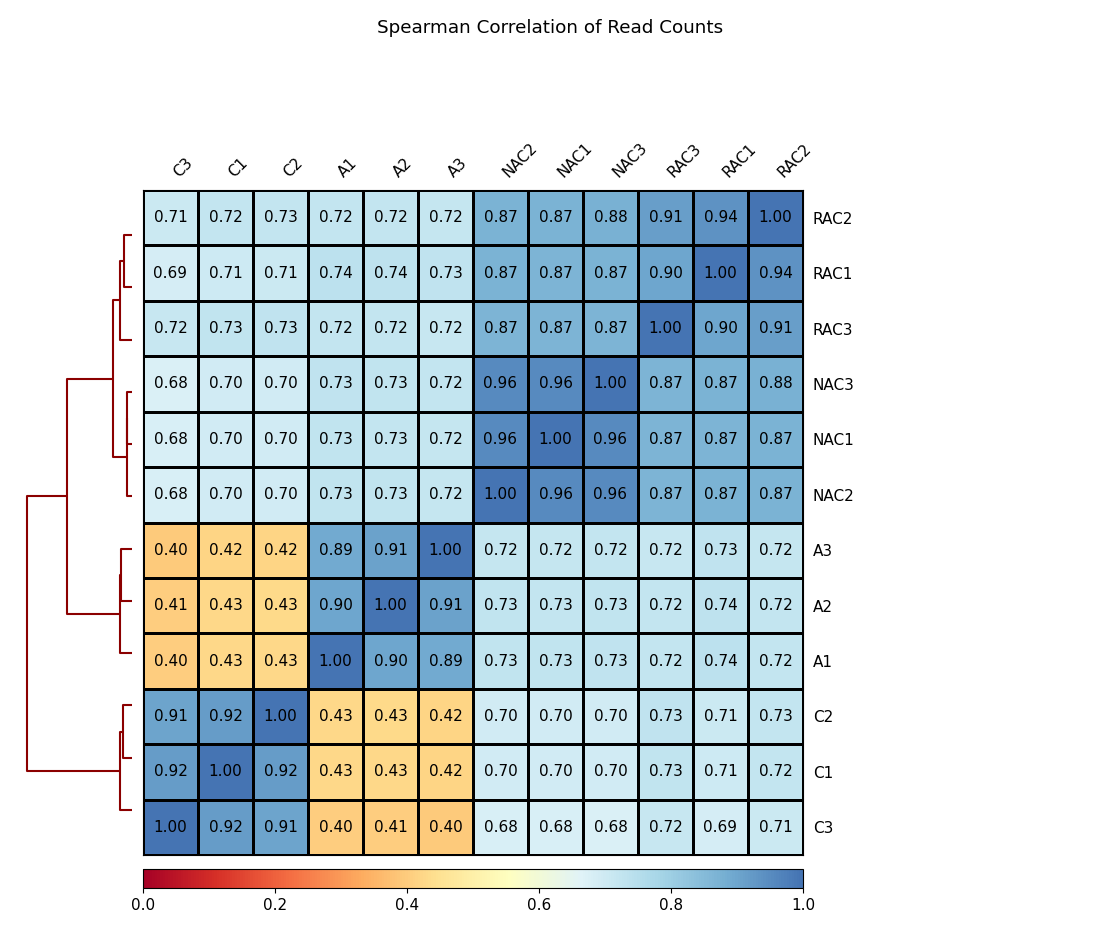

Supplement: Web_Material_uhac230 [file web_material_uhac230.zip › Figure S1.tif]

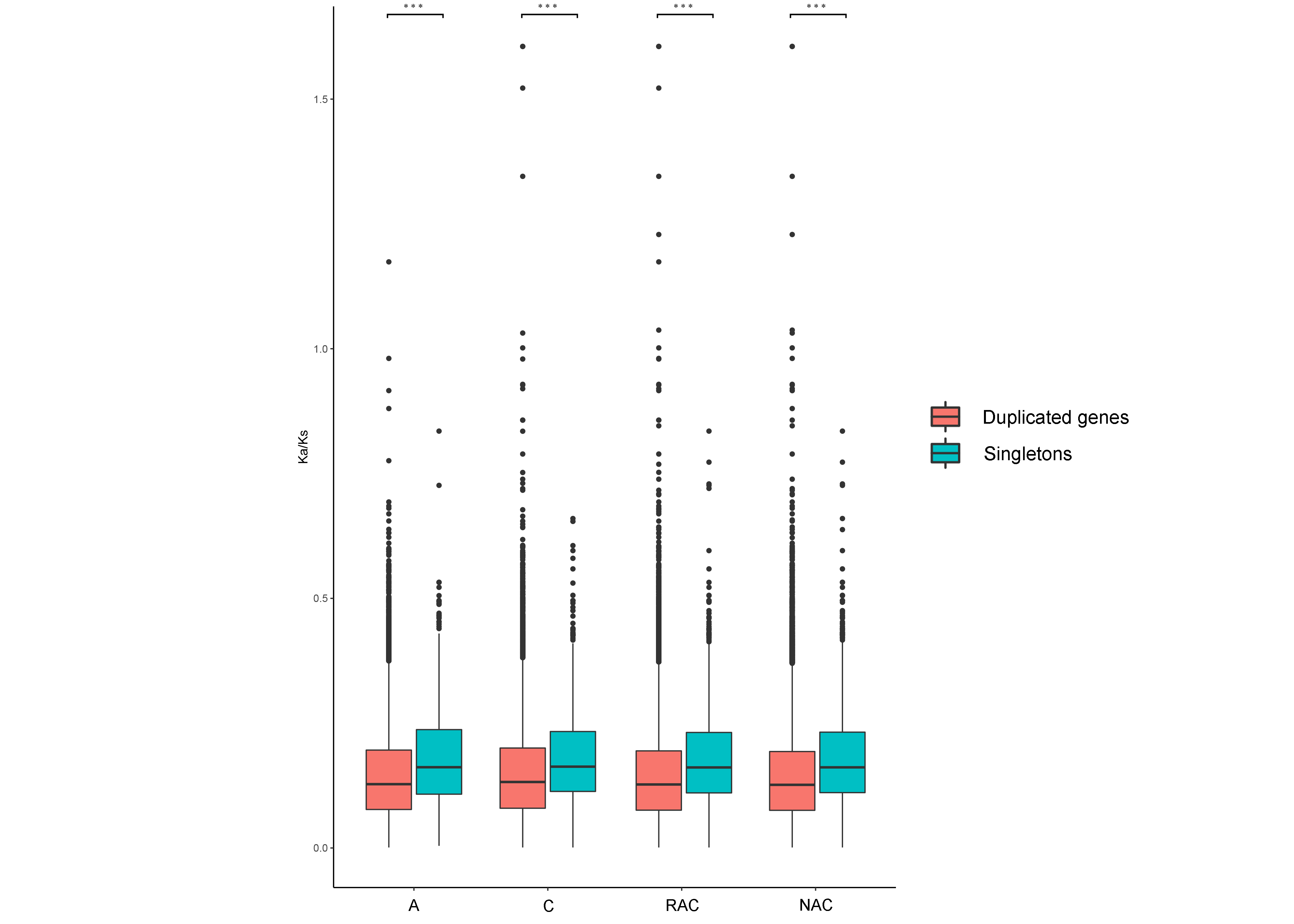

Supplement: Web_Material_uhac230 [file web_material_uhac230.zip › Figure S10.tif]

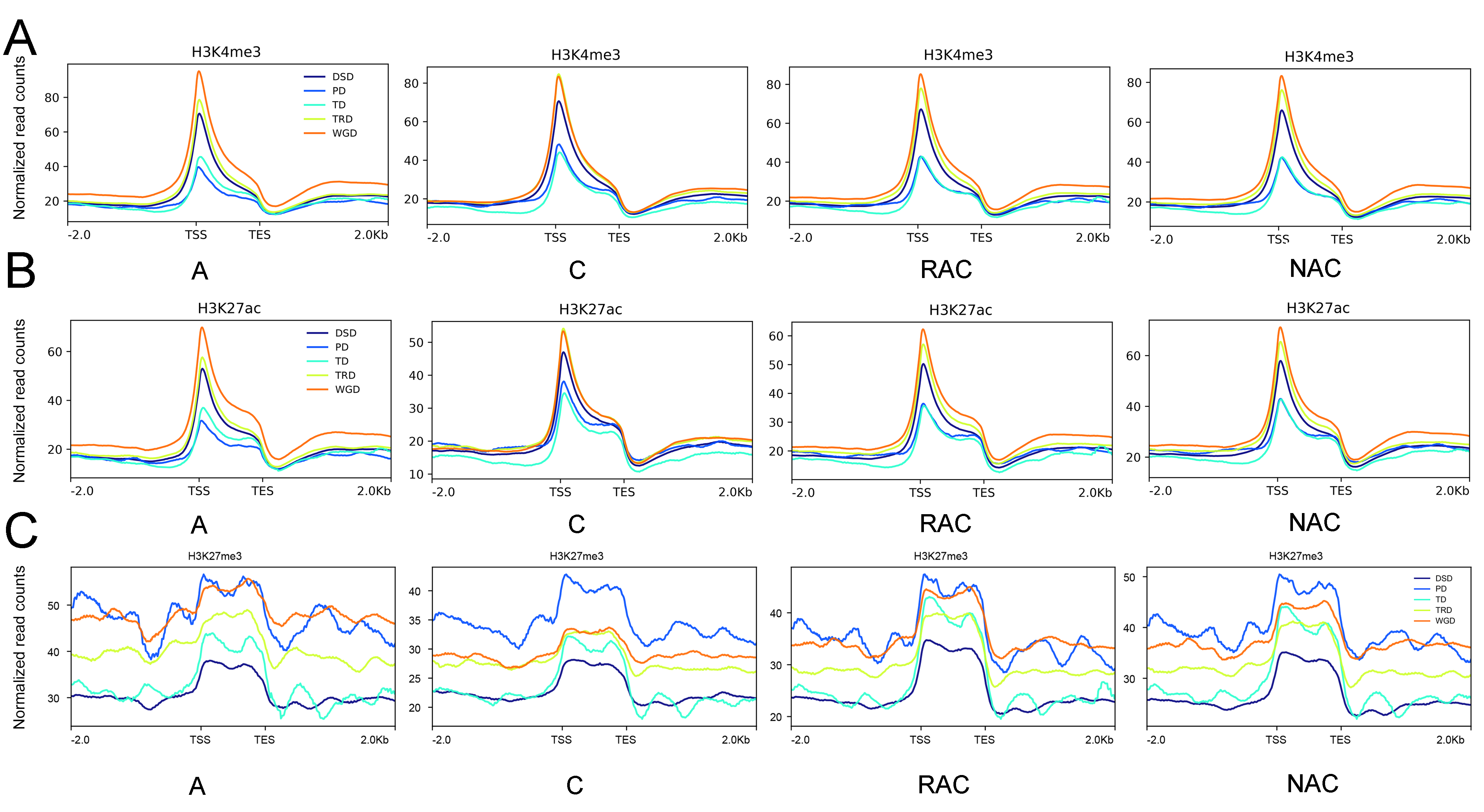

Supplement: Web_Material_uhac230 [file web_material_uhac230.zip › Figure S11.tif]

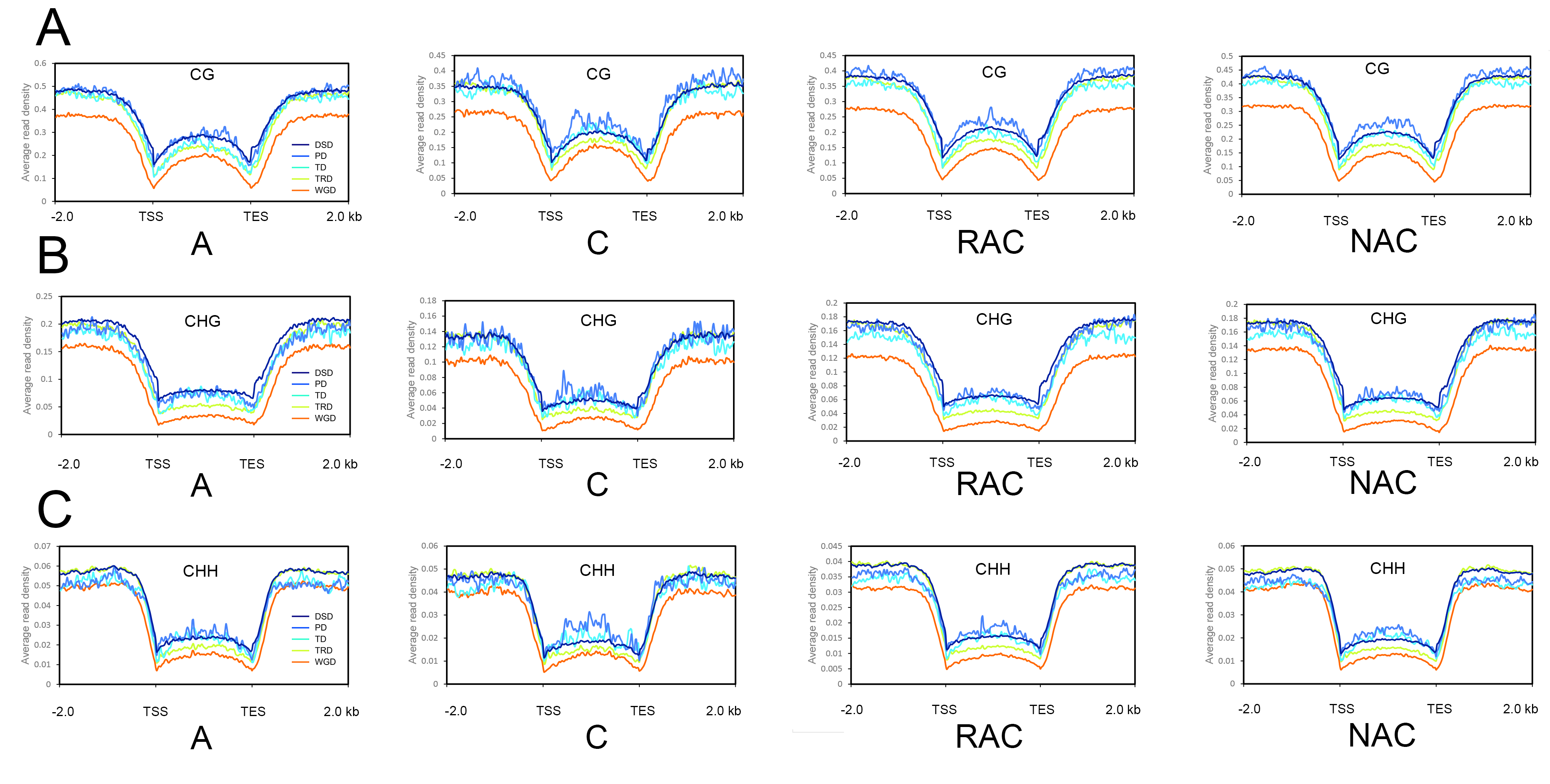

Supplement: Web_Material_uhac230 [file web_material_uhac230.zip › Figure S12.tif]

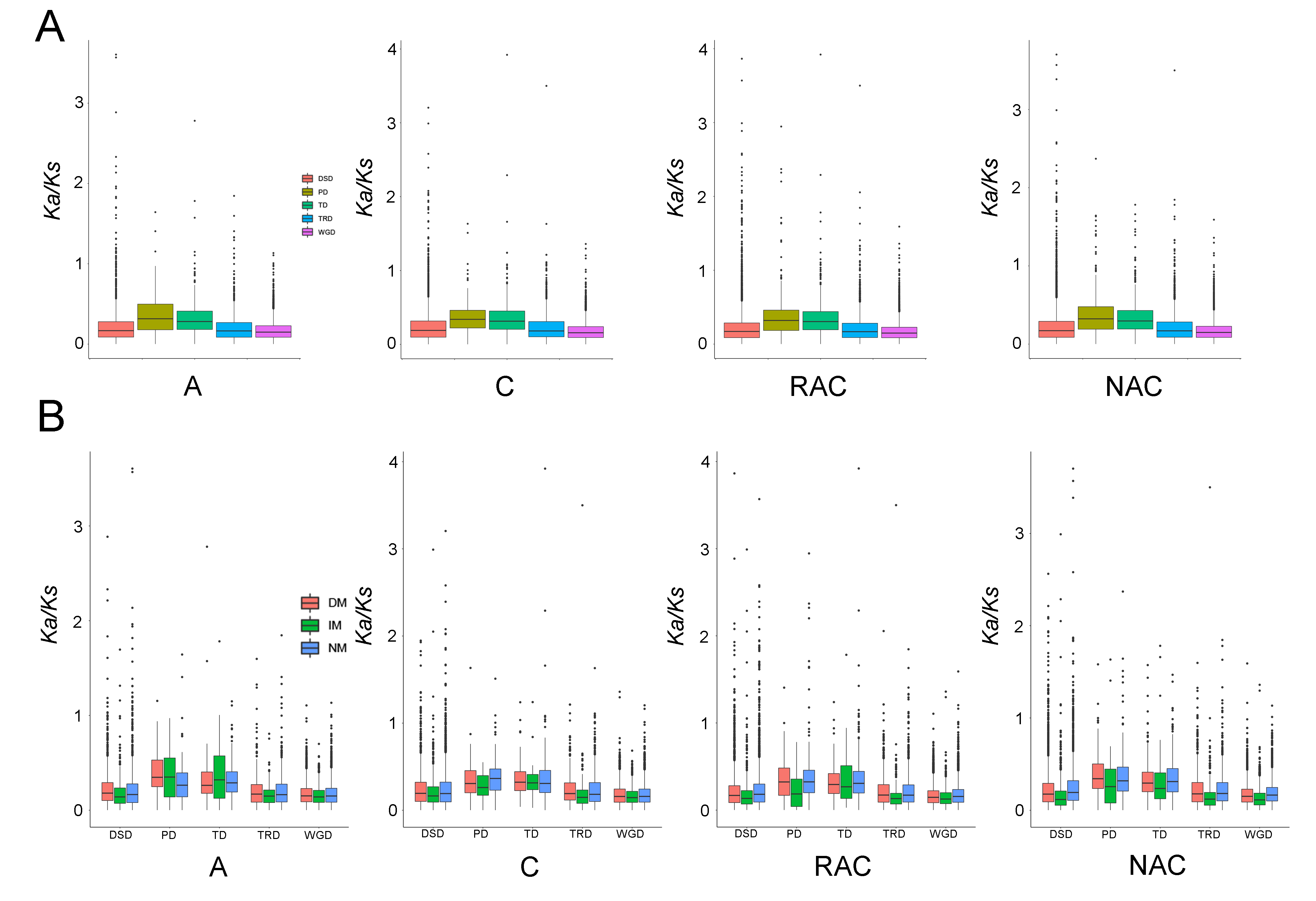

Supplement: Web_Material_uhac230 [file web_material_uhac230.zip › Figure S13.tif]

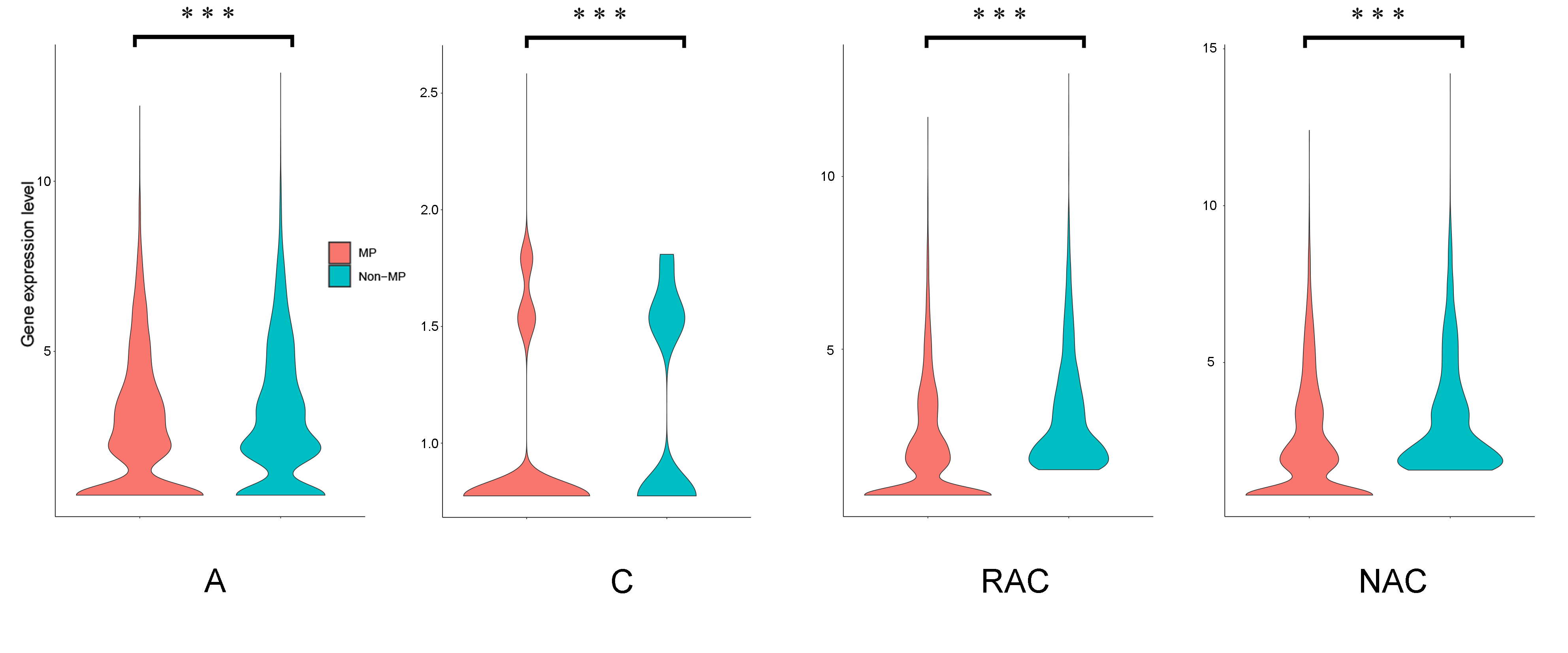

Supplement: Web_Material_uhac230 [file web_material_uhac230.zip › Figure S14.tif]

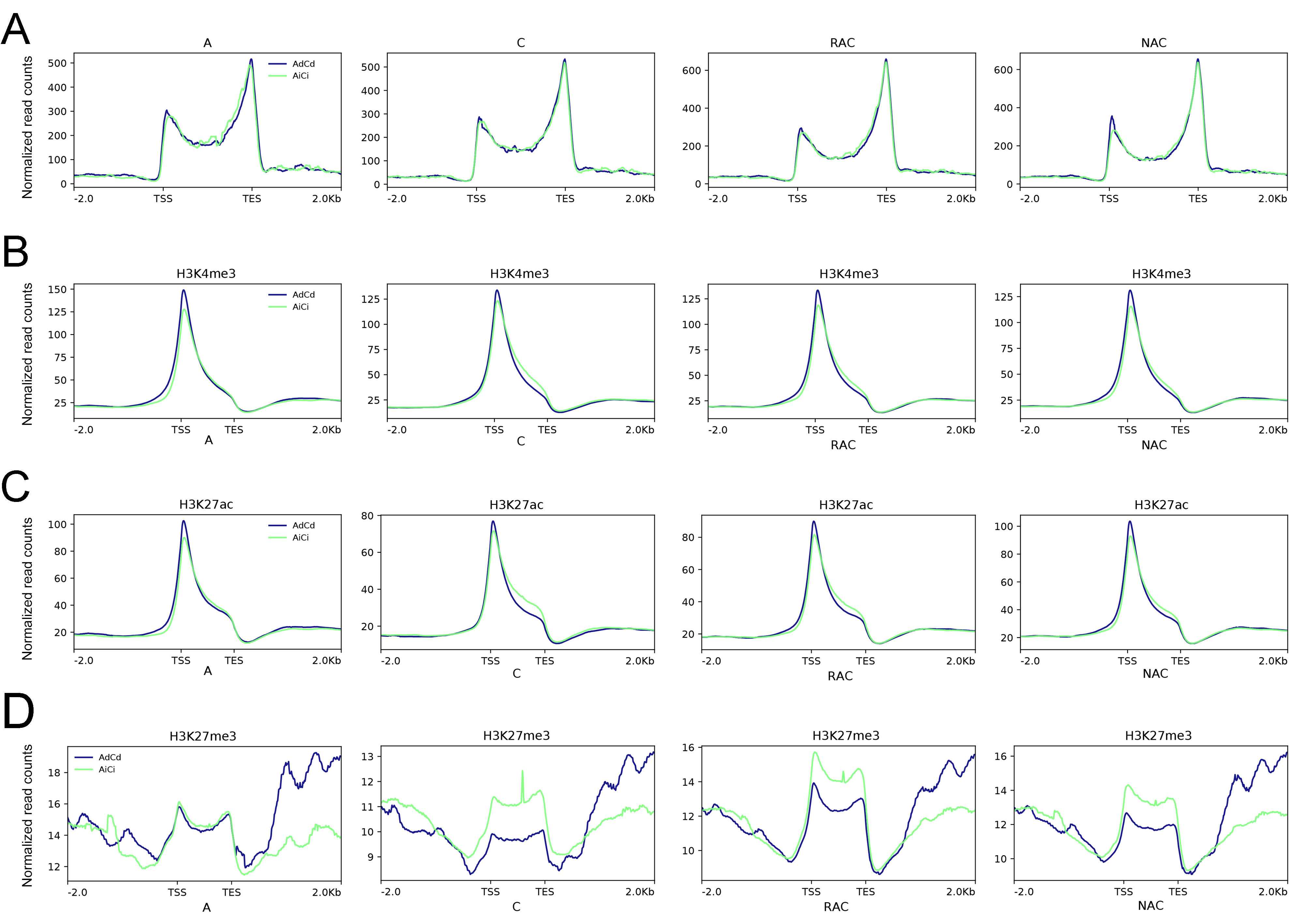

Supplement: Web_Material_uhac230 [file web_material_uhac230.zip › Figure S15.tif]

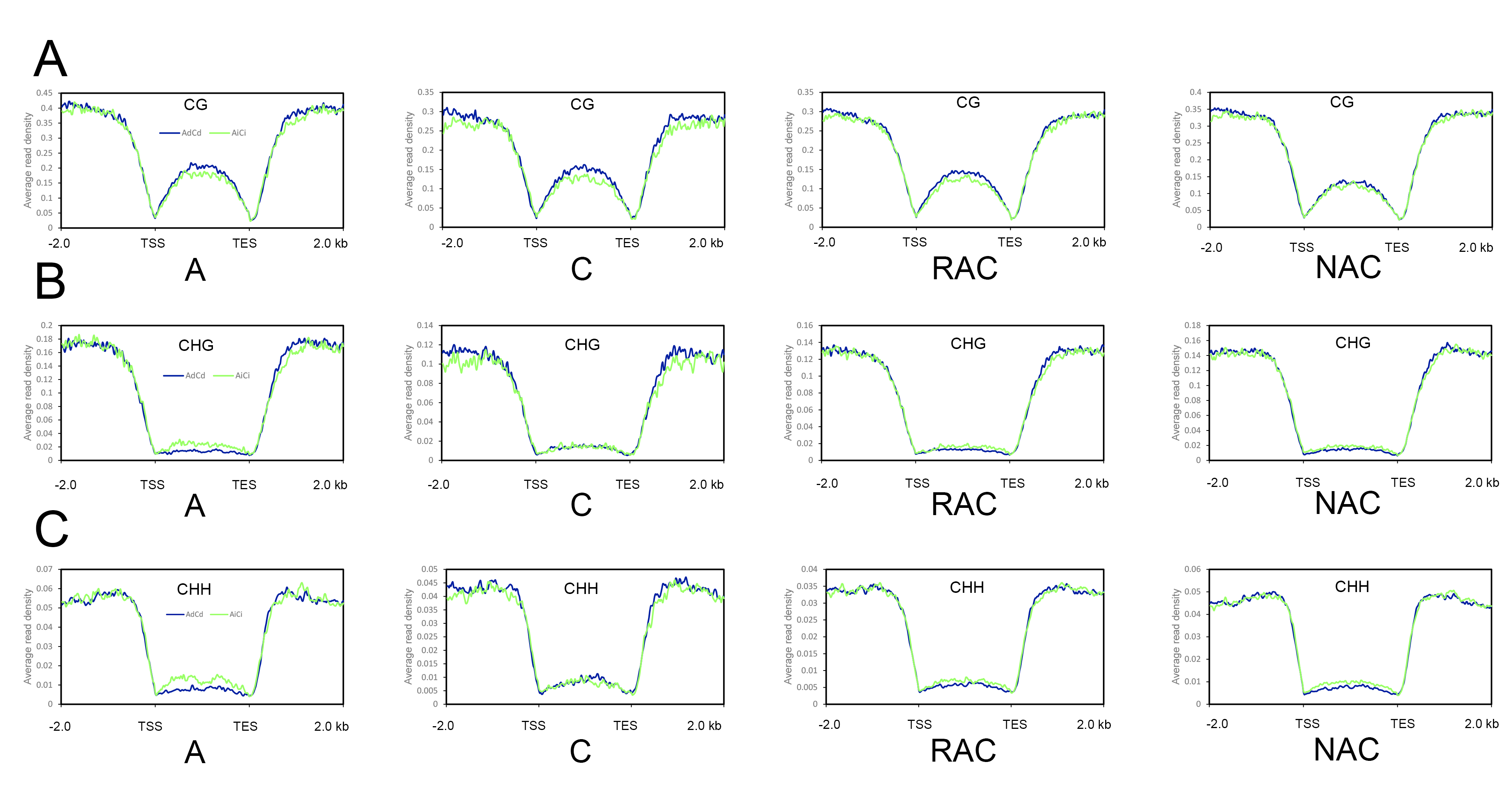

Supplement: Web_Material_uhac230 [file web_material_uhac230.zip › Figure S16.tif]

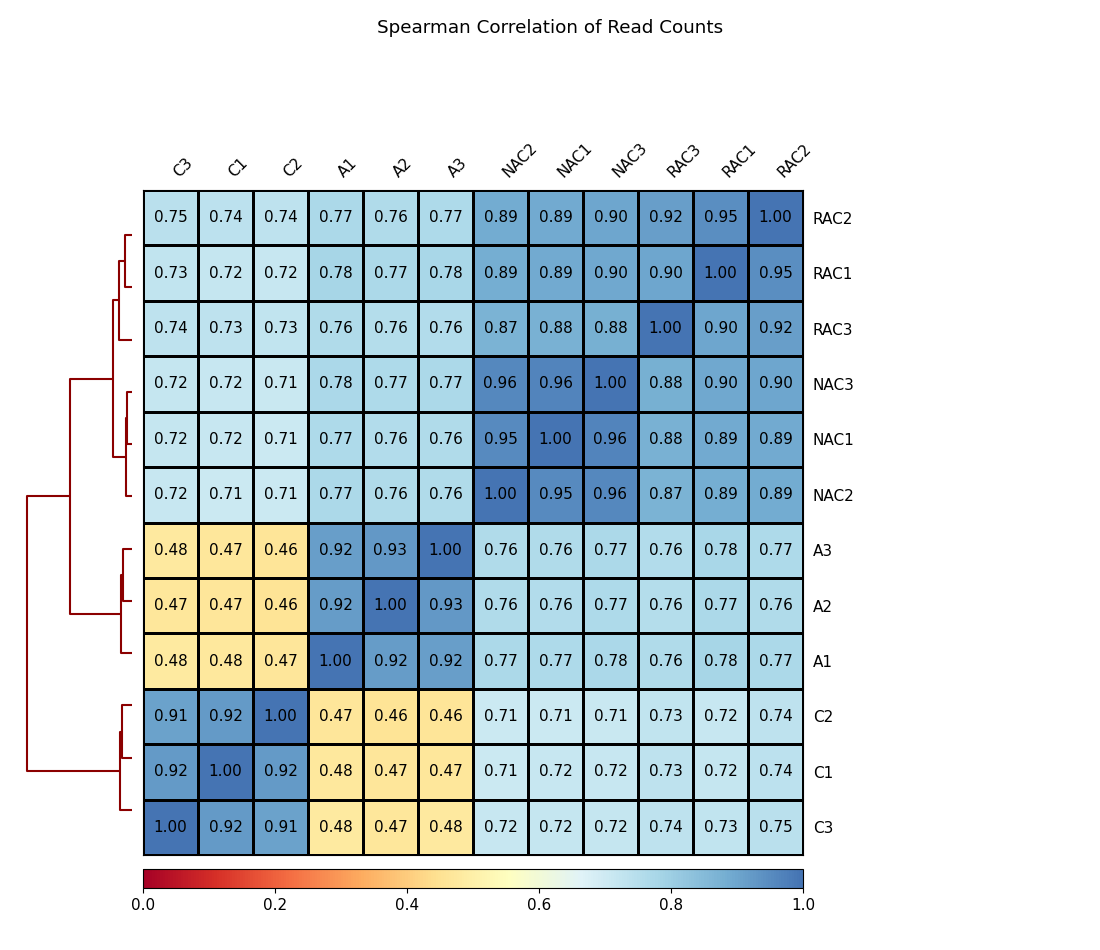

Supplement: Web_Material_uhac230 [file web_material_uhac230.zip › Figure S2.tif]

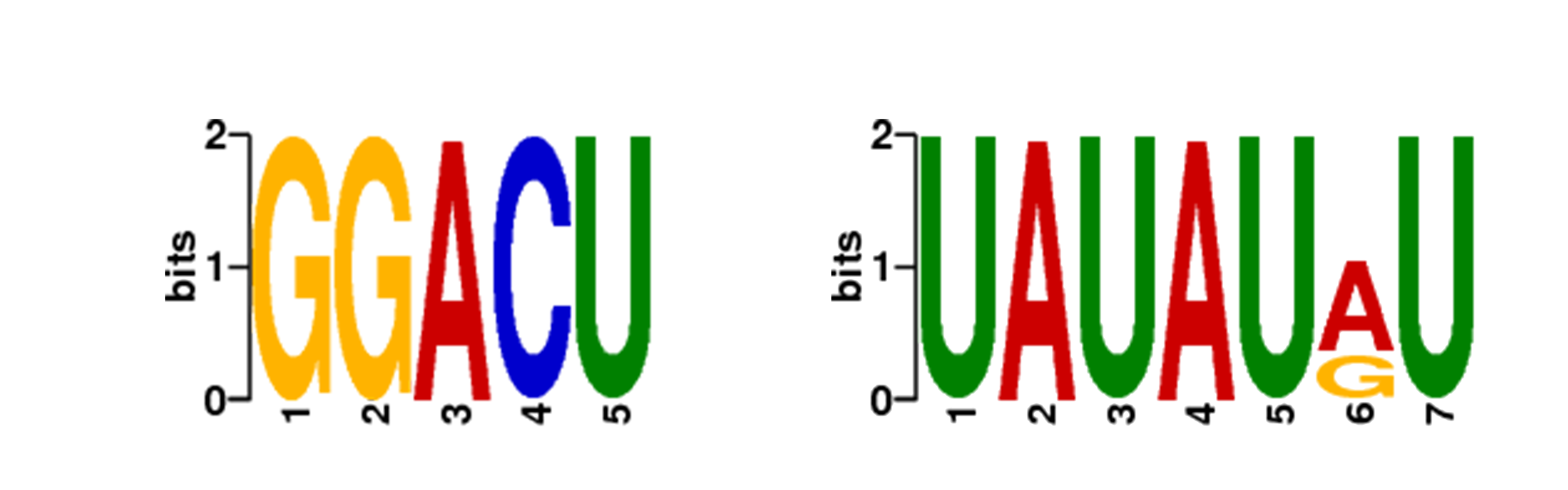

Supplement: Web_Material_uhac230 [file web_material_uhac230.zip › Figure S3.tif]

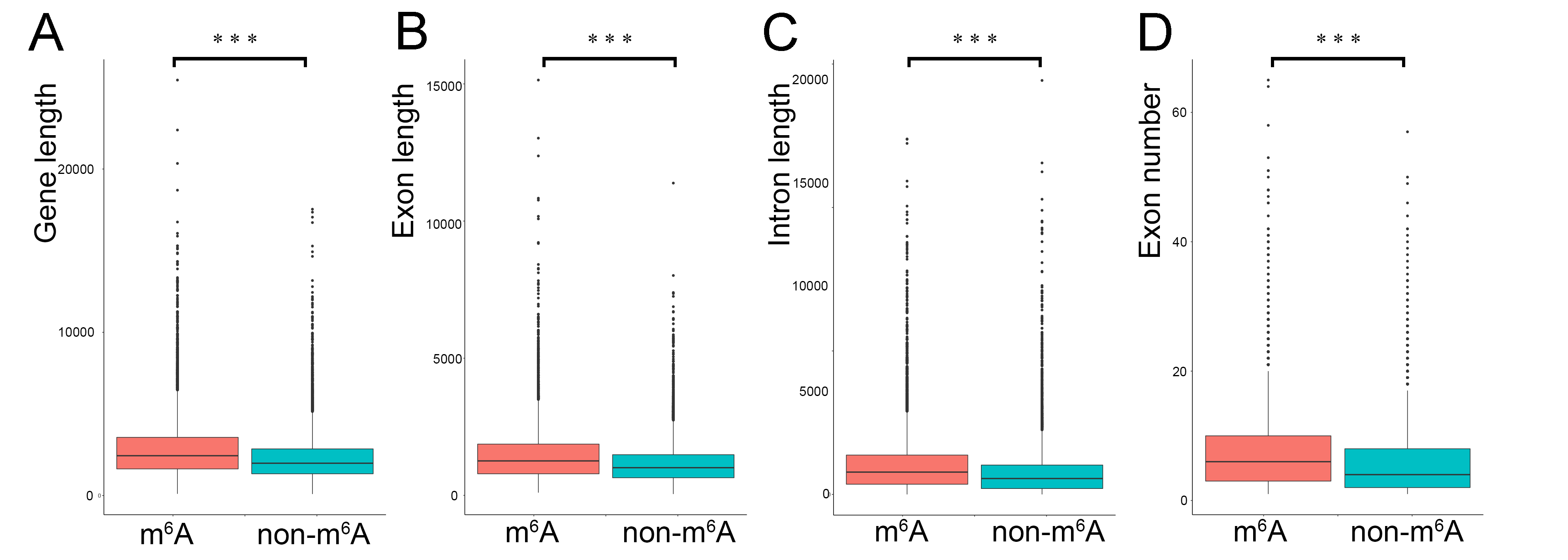

Supplement: Web_Material_uhac230 [file web_material_uhac230.zip › Figure S4.tif]

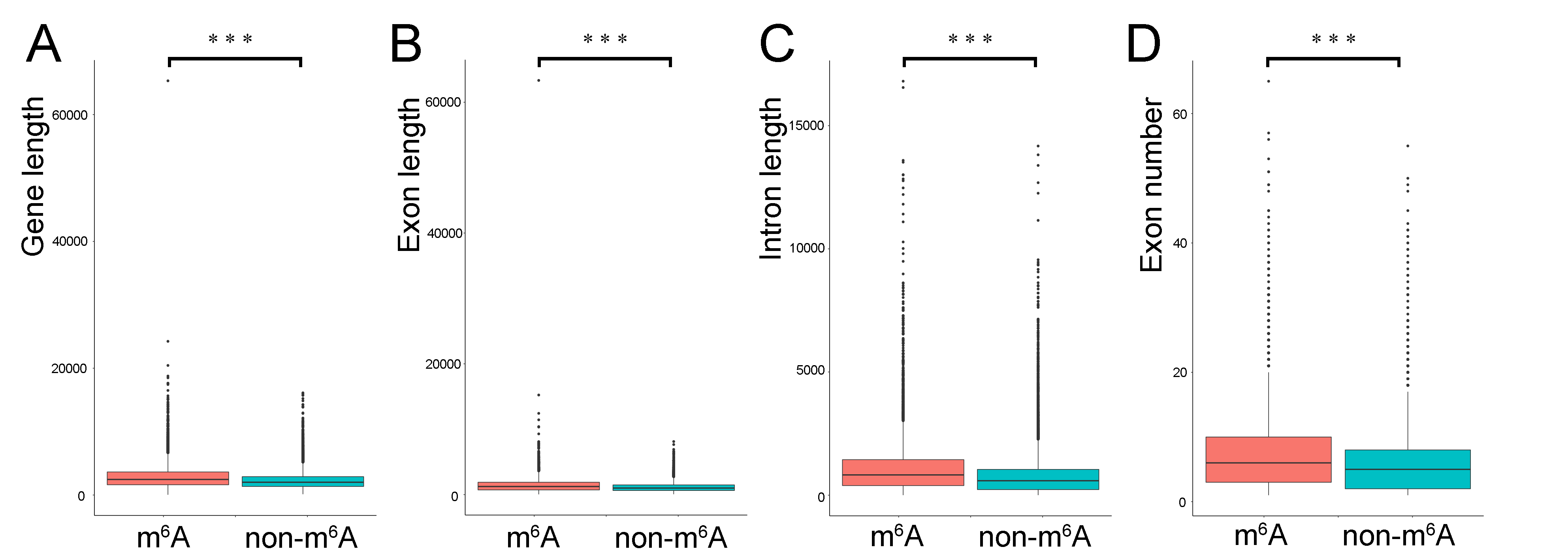

Supplement: Web_Material_uhac230 [file web_material_uhac230.zip › Figure S5.tif]

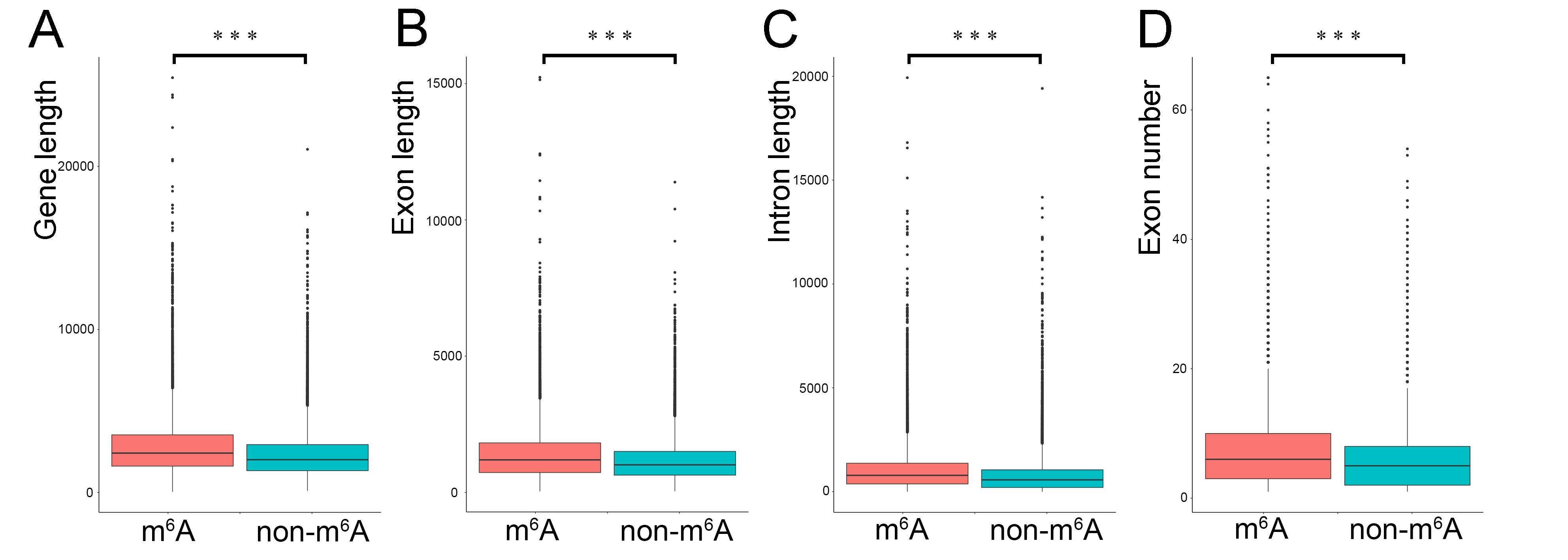

Supplement: Web_Material_uhac230 [file web_material_uhac230.zip › Figure S6.tif]

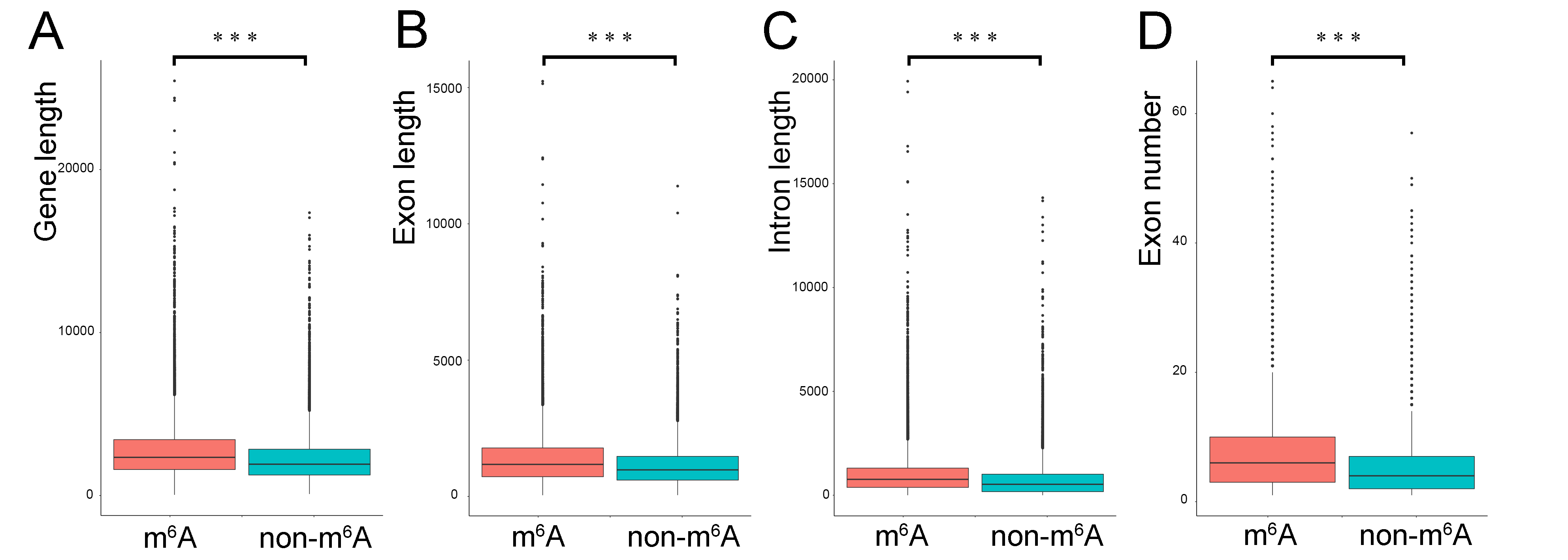

Supplement: Web_Material_uhac230 [file web_material_uhac230.zip › Figure S7.tif]

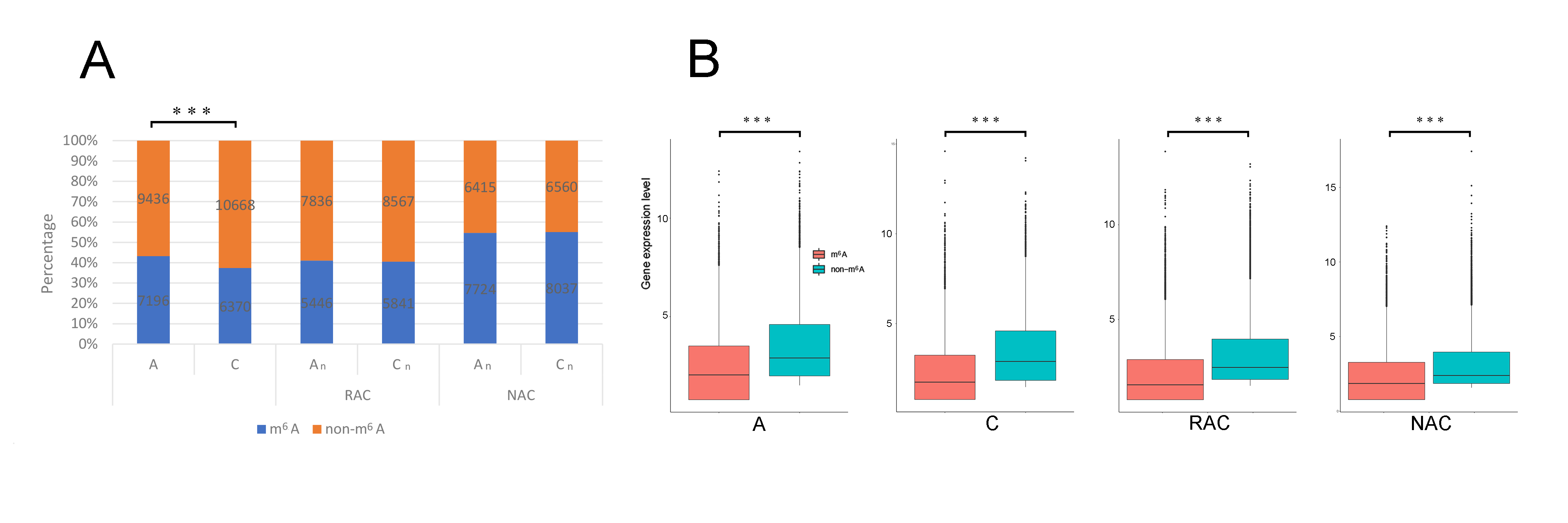

Supplement: Web_Material_uhac230 [file web_material_uhac230.zip › Figure S8.tif]

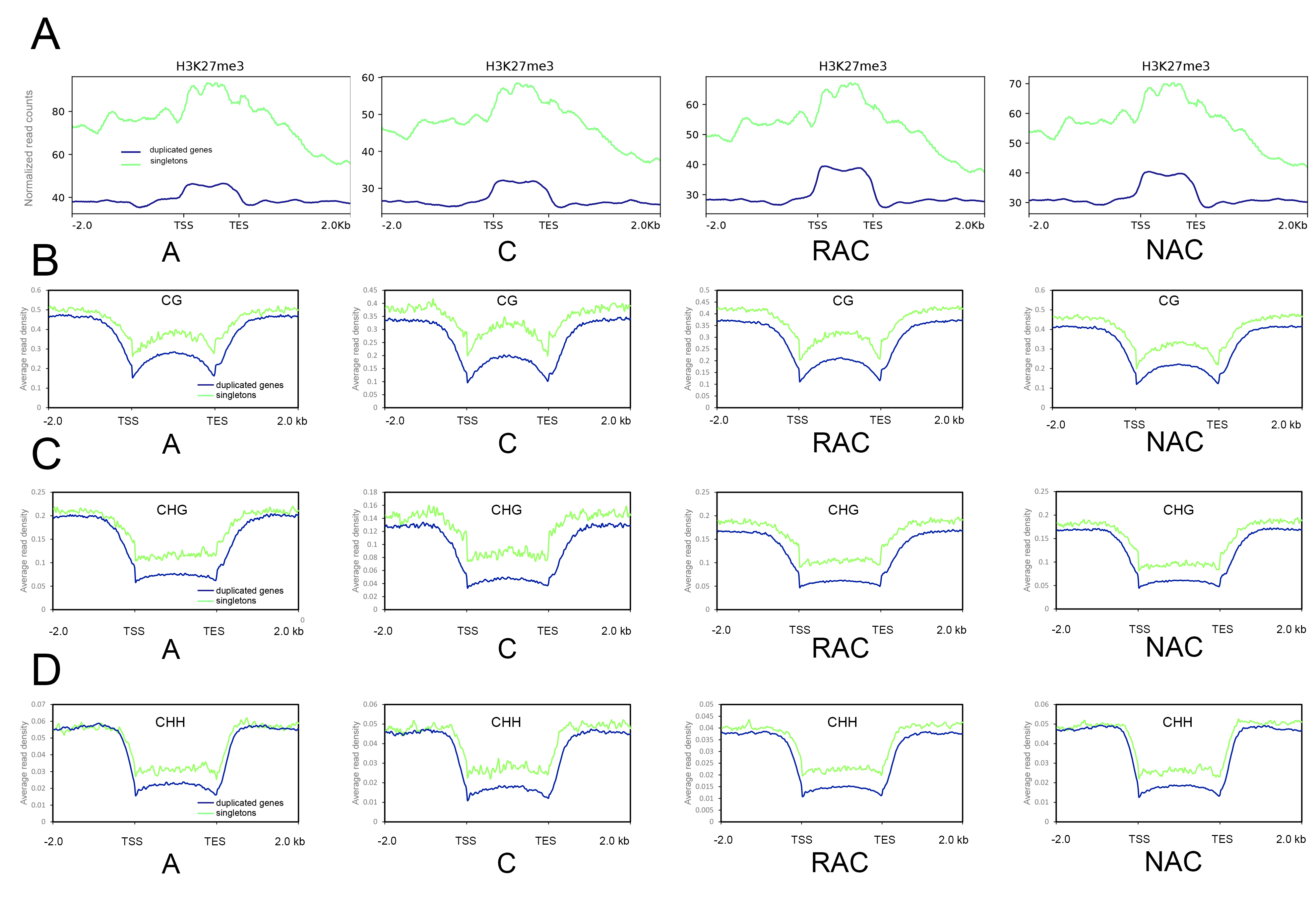

Supplement: Web_Material_uhac230 [file web_material_uhac230.zip › Figure S9.tif]
